# Binding Free Energy Calculation Based on the Fragment Molecular Orbital Method and Its Application in Designing Novel SHP-2 Allosteric Inhibitors

**DOI:** 10.3390/ijms25010671

**Published:** 2024-01-04

**Authors:** Zhen Yuan, Xingyu Chen, Sisi Fan, Longfeng Chang, Linna Chu, Ying Zhang, Jie Wang, Shuang Li, Jinxin Xie, Jianguo Hu, Runyu Miao, Lili Zhu, Zhenjiang Zhao, Honglin Li, Shiliang Li

**Affiliations:** 1 Shanghai Key Laboratory of New Drug Design, State Key Laboratory of Bioreactor Engineering, School of Pharmacy, East China University of Science & Technology, Shanghai 200237, China; y12212121@mail.ecust.edu.cn (Z.Y.); y30211246@mail.ecust.edu.cn (X.C.); y30201249@mail.ecust.edu.cn (S.F.); zhjzhao@ecust.edu.cn (Z.Z.); 2 Innovation Center for AI and Drug Discovery, East China Normal University, Shanghai 200062, China; 3 Lingang Laboratory, Shanghai 200031, China

**Keywords:** binding free energy, fragment molecular orbital method, SHP2

## Abstract

The accurate prediction of binding free energy is a major challenge in structure-based drug design. Quantum mechanics (QM)-based approaches show promising potential in predicting ligand–protein binding affinity by accurately describing the behavior and structure of electrons. However, traditional QM calculations face computational limitations, hindering their practical application in drug design. Nevertheless, the fragment molecular orbital (FMO) method has gained widespread application in drug design due to its ability to reduce computational costs and achieve efficient ab initio QM calculations. Although the FMO method has demonstrated its reliability in calculating the gas phase potential energy, the binding of proteins and ligands also involves other contributing energy terms, such as solvent effects, the ‘deformation energy’ of a ligand’s bioactive conformations, and entropy. Particularly in cases involving ionized fragments, the calculation of solvation free energy becomes particularly crucial. We conducted an evaluation of some previously reported implicit solvent methods on the same data set to assess their potential for improving the performance of the FMO method. Herein, we develop a new QM-based binding free energy calculation method called FMOScore, which enhances the performance of the FMO method. The FMOScore method incorporates linear fitting of various terms, including gas-phase potential energy, deformation energy, and solvation free energy. Compared to other widely used traditional prediction methods such as FEP+, MM/PBSA, MM/GBSA, and Autodock vina, FMOScore showed good performance in prediction accuracies. By constructing a retrospective case study, it was observed that incorporating calculations for solvation free energy and deformation energy can further enhance the precision of FMO predictions for binding affinity. Furthermore, using FMOScore-guided lead optimization against Src homology-2-containing protein tyrosine phosphatase 2 (SHP-2), we discovered a novel and potent allosteric SHP-2 inhibitor (compound **8**).

## 1. Introduction

Computer-aided drug design (CADD) strategies have become an indispensable tool in modern drug discovery and development, with the primary objective of structure-based drug design being the prediction of protein–ligand binding affinity [1,2,3,4]. The accuracy of binding affinity predictions is essential in rationalizing potency, selectivity, and computational drug design. Currently, various strategies have been proposed to predict protein–ligand binding affinities, including low-end empirical scoring functions (SFs) [5], molecular mechanics/Poisson–Boltzmann (generalized Born) surface area (MM/PB(GB)SA) methods [6,7], and theoretically rigorous free energy perturbation (FEP) [8] and thermodynamic integration (TI) [9,10]. However, these force field-based molecular mechanics calculation methods are limited by their ability to accurately capture weak interactions (CH–π, Cl–π, cation–π) in inter- and intra-biomolecules without rigorous testing. In contrast, quantum mechanics (QM) can describe the behavior and structure of electrons and derive the properties of substances [11]. However, applying QM methods toward large biomolecular systems has been computationally quite expensive.

The fragment molecular orbital (FMO) method was proposed by Kitaura et al. [12,13,14,15], and is achieved by dividing the larger system into smaller fragments, which is performed using conventional molecular orbital calculations and offers a substantial computational cost saving over traditional QM methods. This method is helpful in analyzing the interaction energies of the ligand and surrounding residues by calculating fragment–fragment interaction energies. This indicates that the FMO method constitutes a more precise approach than molecular mechanics methods for capturing weak interactions. Therefore, this method has a wide range of applications in the field of drug design, including structure-based drug design (SBDD) [16,17,18,19], fragment-based drug design (FBDD) [20], and protein–protein interactions (PPIs) [21,22,23,24]. In structure-based drug design, the FMO method proves highly valuable for the quantitative understanding of molecular interactions. Medicinal chemists and computational chemists employ interaction data between ligands and individual residues of the protein to engage in structure–activity relationship research, establishing a theoretical foundation for ligand design [25,26]. This information is also instrumental in predicting binding modes [27,28], performing virtual screening [29,30,31], and elucidating molecular recognition mechanisms within RNA–ligand systems [32], among other applications.

While FMO calculations represent a valuable approach for enhancing enthalpic contributions in drug design, the phenomenon of protein–ligand binding is an exceedingly complex process where binding might be driven by different energy terms, including direct enthalpic contributions, entropy, solvation effects, and the ‘deformation energy’ of the ligand’s bioactive conformation [33]. Solvation effects should be taken into consideration when assessing binding affinities, particularly in cases involving interactions with ionized fragments [34]. In recent years, solvation methods have been incorporated into the FMO method through the utilization of implicit solvent models such as the polarized continuum model (PCM) and solvation model density (SMD) [35,36,37,38,39]. In addition, the solvation energies were calculated in separate calculations using generalized Born/surface area (GBSA) or the Poisson–Boltzmann/surface area (PBSA) method [40,41,42,43]. These solvation methods were anticipated to enhance the precision of FMO binding affinity calculations, and previous studies have reported their efficacy [44,45,46]. 

In the context of the large-scale systems encountered in drug design, semi-empirical quantum mechanical methods offer a compromise between time and precision. The PM7/COSMO solvation model demonstrates good performance in calculating solvation energy changes during binding [47]. Hence, we intend to assess the performance of three levels of implicit solvent models (ab initio, semi-empirical, and molecular mechanics (MM)-based) on FMO binding affinity calculations using the Schrödinger FEP+ data set [48] as a benchmark. Currently, studies have reported that the incorporation of ligand deformation energy can enhance the predictive performance of the FMO /PCM method in binding affinity predictions [36,49]. However, as of now, there is no reported research on the contribution of entropy. Hence, we explore the optimal combinations of enthalpy of FMO calculation, solvation free energy, deformation energy, and entropy to deal with a wide variety of drug design cases.

In this work, we develop an FMO-based binding affinity calculation method (FMOScore) with linear fitting on terms including solvation free energy, calculated by the PM7/COSMO model [50], and the deformation energy term of ligands. When the entropy term, calculated with the interaction entropy (IE) method [51], was added, FMOScore exhibited a slight performance boost. Considering time consumption and cost computationally, we discarded the entropy term. The performance study of FMOScore was carried out and compared with other methods of calculating the free energy of binding (FEP+, MM/PB(GB)SA, Vina) on the Schrödinger FEP+ benchmark of eight targets containing 258 complexes. Results demonstrated that FMOScore improved the prediction accuracy of the FMO method and displayed better or equally high correlation to the experimental data compared to the results for these traditional methods (FEP+, MM/PB(GB)SA, Vina).

Further, FMOScore was applied to design new inhibitors against Src homology-2-containing protein tyrosine phosphatase 2 (SHP-2), which is a non-receptor protein tyrosine phosphatase encoded by the gene *PTPN11* [52]. SHP-2 is recognized as a promising drug target for the treatment of multiple cancer types and tumors, including leukemia, Noonan syndrome (NS), LEOPARD syndrome (LS), colon cancer, melanoma, lung adenocarcinoma, neuroblastoma, and hepatocellular carcinoma [53,54,55]. Previously, SHP-2 inhibitors targeting the catalytic PTP domain failed to advance successfully into clinical trials mainly because of their drawbacks, including poor membrane permeability, low selectivity, and poor oral bioavailability [56]. To overcome these shortcomings, Novartis reported the first allosteric inhibitor, SHP099 (SHP-2^WT^ IC_50_ = 0.071 μM), with high selectivity and low toxicity that stabilizes the auto-inhibited conformation of SHP-2 at the “tunnel” allosteric site [57]. Until now, several SHP-2 allosteric inhibitors, such as JAB-3068, JAB-3312, TNO155, ERAS-601, RMC-4630, BBP-398, SH3809, RLY-1971, and HBI-2376, have advanced into clinical trials for cancer treatment [58]. In addition, recent studies have reported that SHP-2 inhibitors, activators, and proteolysis-targeting chimera (PROTAC)-based degraders also display therapeutic promise [59,60,61]. Herein, we report strategies for scaffold hopping and structural modifications based on a lead compound (SHP099) using the FMOScore method, leading to the discovery of compound **8** as a novel and potent allosteric SHP-2 inhibitor. These encouraging outcomes suggest that FMOScore may be valuable in various structure-based drug design efforts for rational drug discovery.

## 2. Results

### 2.1. Influence of Energy Terms on the Performance of FMOScore

**Figure 1:**
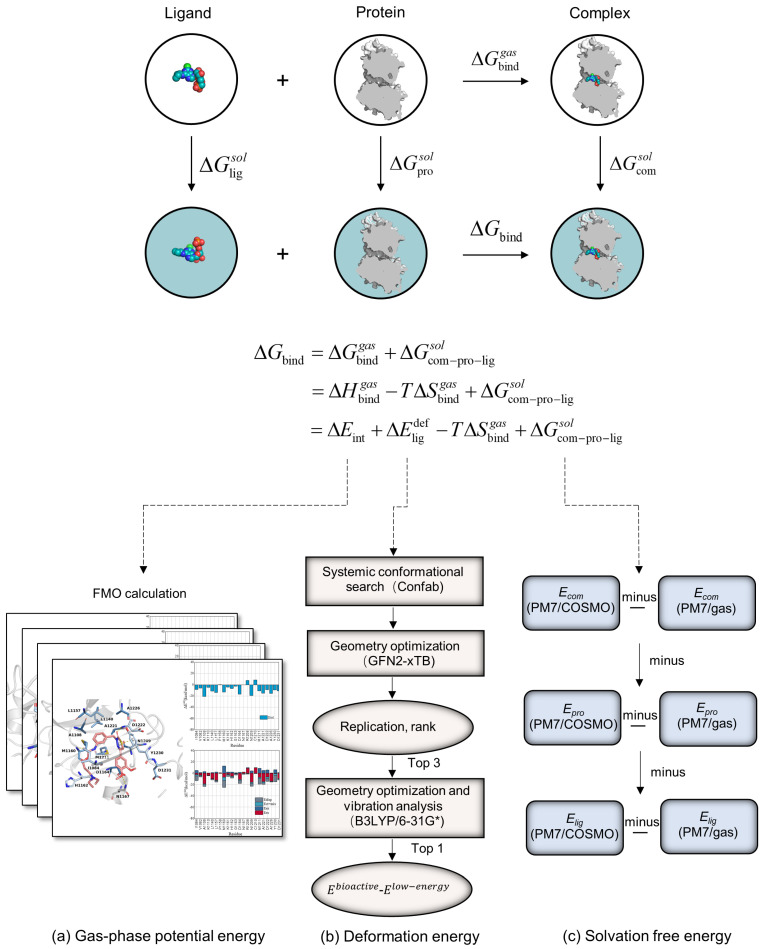
Computational workflow of the FMOScore approach, which contains three terms: (**a**) gas-phase potential energy, (**b**) deformation energy, and (**c**) solvation free energy.

**Figure 2:**
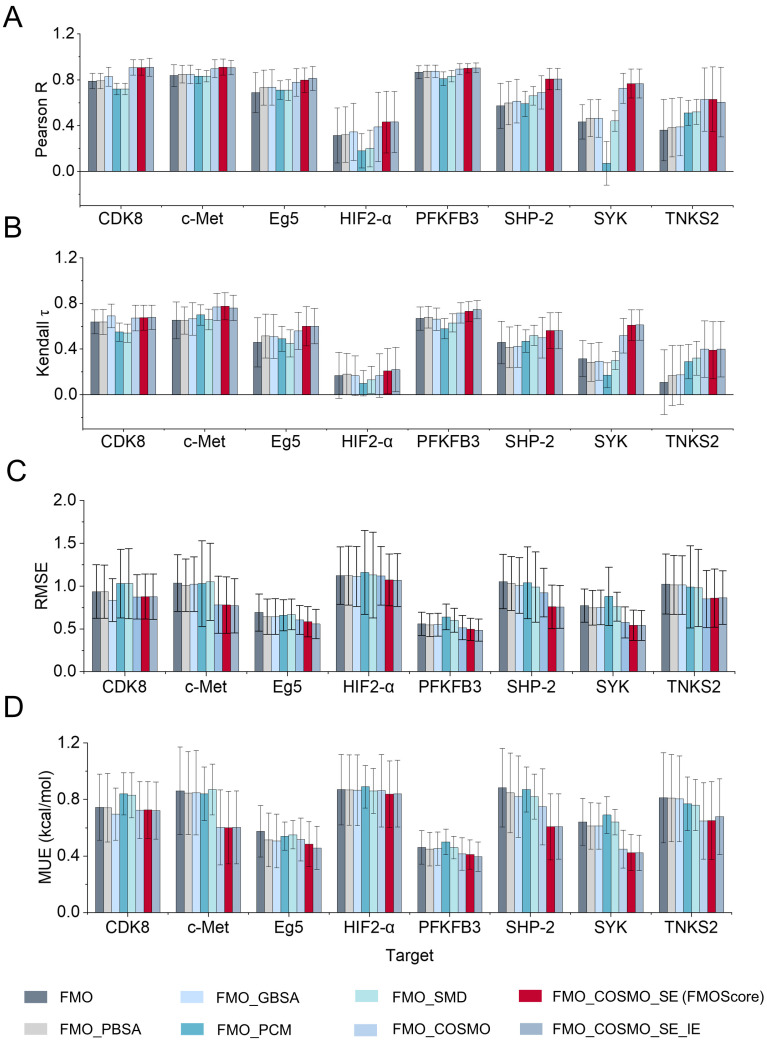
Performance of 8 functions for each of 8 targets in Schrödinger FEP+ benchmark. (**A**) Pearson correlation coefficient *R*. (**B**) Kendall *τ*. (**C**) Root-mean-squared error (RMSE). (**D**) Mean unsigned error (MUE). The error bars indicate the 90% confidence interval by bootstrap with 10,000 resampling events.

Given the thermodynamic cycle, free energy can be split into several components, including the gas-phase potential energy, solvation free energy, and the entropy of ligand–receptor interactions (Figure 1). Therefore, we constructed an FMO-based binding affinity prediction strategy, which consisted of eight functions (Table 1). These functions included the calculation of the solvation free energy term by the PBSA model (function form 2: FMO_PBSA), the GBSA model (function form 3: FMO_GBSA), the PM7/COSMO model (function form 4: FMO_COSMO), the PCM model (function form 5: FMO_PCM), and the SMD model (function form 6: FMO_SMD). Additionally, the deformation energy term of ligands was calculated (function form 7: FMO_COSMO_SE), and the entropy term was calculated by the IE method (function form 8: FMO_COSMO_SE_IE). Detailed linear coefficients are given in Table S2. Table 1 summarizes the functions’ integral performance to the eight biological targets in the Schrödinger FEP+ benchmark [48] (confidence intervals calculated by bootstrap sampling for each data set). The statistics of each target for Pearson correlation coefficient *R*, Kendall τ, the root-mean-squared error (RMSE), as well as the mean unsigned error (MUE), are also shown in Figure 2.

To explore the best-fit function of the FMO-based binding affinity calculation model, the Schrödinger FEP+ benchmark was used to evaluate its prediction power. We performed linear fitting on the calculated energy terms and experimental binding affinities of eight targets, respectively. The results showed that the binding affinities using the conventional FMO method on this data set exhibited a relatively low correlation with the experimental values (average Pearson *R* = 0.$62_{0.68}^{0.55}$, average Kendall *τ* = 0.$47_{0.52}^{0.41}$). The implicit solvation models (PBSA, GBSA, COSMO, PCM, SMD) were used to calculate the solvation free energies that evaluated the response of the whole solute energy. The PM7/COSMO model systematically improved the correlation between the scores and experimental binding free energies, with a 37.1% improvement of the Pearson *R* and a 35.0% decrease in the averaged RMSE over the FMO method. As expected, the semi-empirical quantum mechanical (SQM) method, the PM7/COSMO solvation model, was the best-performing method, which had a higher accuracy than the PBSA or GBSA models. This improvement can be mainly attributed to the algorithmic simplicity, numerical stability, and great insensitivity for outlying charge errors by the PM7/COSMO model [62]. It is noteworthy that the performance of function form 3 (FMO_GBSA) was slightly better than that of function form 2 (FMO_PBSA). Previous studies have also reported that molecular mechanics/ Generalized-Born surface area (MM/GBSA) performed better than molecular mechanics/Poisson-Boltzmann surface area (MM/PBSA) for both binding pose predictions and binding free energy estimations [41]. In addition, we separately incorporated both PCM and SMD implicit solvent models into the FMO method to assess binding affinity. The results indicated that the binding affinities using the SMD solvent effects marginally improved the correlation with experimental values, with a 17% increase in Pearson *R*. However, the binding affinity predictions using the PCM solvation effects did not exhibit a good correlation with experimental values (average Pearson *R* = 0.$43_{0.60}^{0.26}$). This could possibly be attributed to the poor performance of the PCM for specific protein cavities, resulting in a lack of benefit from the nonpolar contributions to protein–ligand binding; on the contrary, the results deteriorate [35,63,64]. Because of the COSMO method used in our scoring function, the computational cost was much lower than the combination of traditional FMO calculations with the PCM or the SMD solvent model. 

Hence, we added the deformation energy of the ligand into function form 4 (FMO_COSMO) to obtain function form 5 (FMO_COSMO_SE). The performance of the model increased significantly (Pearson *R* increased by 40.3% and RMSE decreased by 37.7%, compared to function form 1). When the entropy term was included in function form 6 (FMO_COSMO_SE_IE), the prediction power was slightly elevated compared to that in function form 5 (FMO_COSMO_SE). Considering the calculation of the entropy term involves MD simulation sampling and the extraction of protein–ligand interactions from a trajectory of 100,000 frames, it incurs a high computational cost; hence, we chose function form 5 (FMO_COSMO_SE) as the FMO-based binding affinity prediction model (called FMOScore) in this work. 

### 2.2. Prediction Performance of the FMOScore Approach

**Figure 3:**
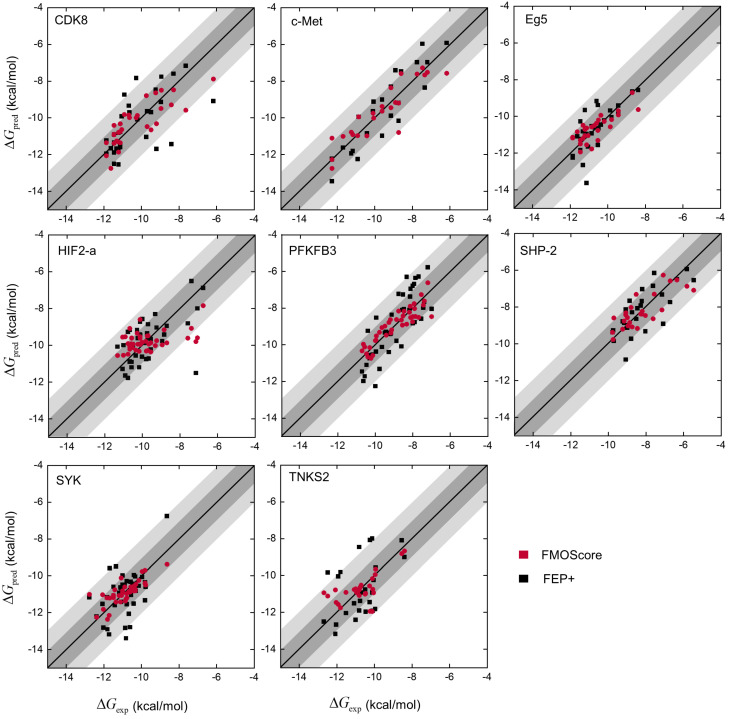
Prediction results of FMOScore in the Schrödinger FEP+ benchmark. Δ*G* values are predicted by FMOScore in red dots and FEP+ in dark dots. Predictions within 1 and 2 kcal/mol of the experimental affinity are highlighted by dark and light gray areas, respectively.

Achieving absolute accuracy in binding affinity predictions is crucial to provide definitive guidance for lead optimization. Therefore, a performance assessment of FMOScore developed was carried out, whose results are summarized in Table S1 (detailed plots for the per-target data set can be found in Figure 3). In seven data sets (CDK8, c-Met, EG5, PFKFB3, SHP-2, SYK, and TNKS2), FMOScore demonstrated an excellent ability to predict binding free energy (Pearson *R* > 0.62, Kendall *τ* > 0.5, RMSE < 0.9 kcal/mol). In particular, FMOScore produced a good ranking in the c-Met and PFKFB3 data sets (0.$77_{0.88}^{0.65}$ and 0.$73_{0.81}^{0.64}$ by Kendall τ, respectively). Meanwhile, the experimental and predicted free-energy changes (ΔΔ*G*), presented as box-plot diagrams in Figure S1, indicated the high prediction accuracy of FMOScore, which measured accuracy by comparing predicted free energy Δ*G*_pred_ to experimental affinity Δ*G*_exp_ for each compound individually. Except for the HIF-2α target, the predicted Δ*G*_pred_ was within 2 kcal/mol of the experimental value using the FMOScore method in seven data sets (Figure 3 and Figure S1). However, the predictive performance of FMOScore is poor for the HIF-2α target, possibly due to the pivotal role of entropy contributions in ligand binding, while FMOScore primarily focuses on the precise calculation of enthalpy contributions. Despite our attempts to enhance the performance of the FMOScore method by employing the IE method for entropy calculation, favorable outcomes were not achieved.

Moreover, the contributions of different energy terms for the Schrödinger FEP+ benchmark calculated by FMOScore are summarized in the Supplemental Data, where the pair interaction energy calculated with the FMO method can be decomposed (PIEDA). The PIEDA analysis decomposes the interaction energy into the electrostatic and charge transfer terms, the exchange-repulsion term, and the dispersion term, which describe H-bonds (or salt bridges, polar interactions), steric repulsion, and hydrophobic interactions. Figure S2 and Table S3 display detailed information on the individual contribution of each residue to the lead compound of the eight targets and provide a reference for the rational structure-based drug design (SBDD) strategy.

### 2.3. Comparison with Traditional Methods for Calculating the Binding Free Energy

**Figure 4:**
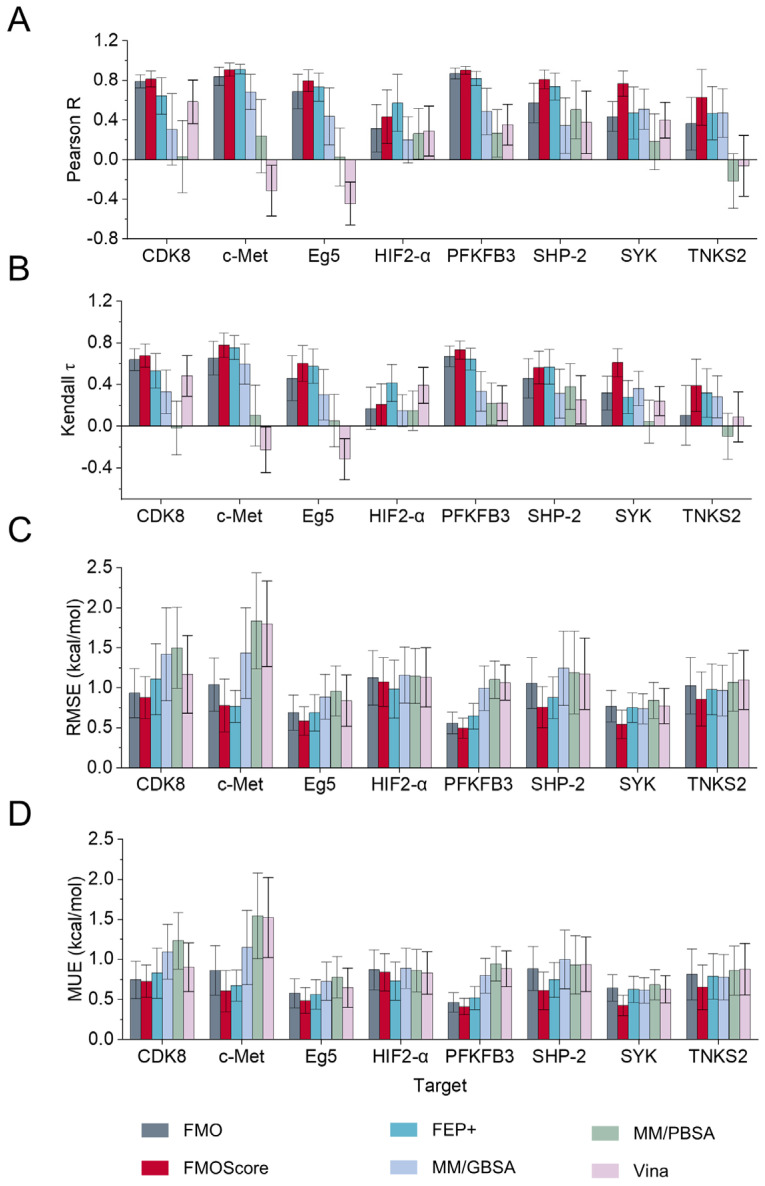
Performance comparison of FMOScore and traditional binding free energy calculation methods. (**A**) Pearson correlation coefficient *R*. (**B**) Kendall τ. (**C**) Root-mean-squared error (RMSE). (**D**) Mean unsigned error (MUE). The error bars indicate the 90% confidence interval by bootstrap with 10,000 resampling events.

The primary goal of FMOScore is to predict the binding free energy of a given protein–ligand complex. Comparing FMOScore results on the benchmark with FEP+, MM/GBSA, MM/PBSA, and Vina, we found that FMOScore performed better than these traditional structure-based drug design (SBDD) methods (Figure 4 and Table S1). In detail, the FMOScore method achieved better correlation (average Pearson *R* = 0.$87_{0.89}^{0.84}$) and higher-ranking power (average Kendall *τ* = 0.$69_{0.72}^{0.65}$) than FEP+ (average *R* = 0.$78_{0.83}^{0.74}$, average Kendall *τ* = 0.$58_{0.63}^{0.53}$). Among the eight data sets tested, it exhibited comparable performance in binding free energy prediction of FMOScore and FEP+ (Figure 3 and Figure 4 and Table S1). In addition, the overall performance of FMOScore (average Pearson *R* = 0.$87_{0.89}^{0.84}$, average RMSE = 0.$71_{0.80}^{0.62}$ kcal/mol) was better than that of MM/GBSA (average Pearson *R* = 0.$27_{0.36}^{0.18}$, average RMSE = 1.$39_{1.54}^{1.25}$) and MM/PBSA (average Pearson *R* = 0.$13_{0.23}^{0.03}$, average RMSE = 1.$44_{1.57}^{1.30}$), and was much better than that of Autodock Vina (average Pearson *R* = 0.$09_{0.18}^{0.008}$, average RMSE = 1.$44_{1.57}^{1.30}$) (Figure 4 and Table S1). Therefore, this confirmed that FMOScore has higher prediction accuracy on this benchmark consisting of eight data sets with structurally diverse sets of ligands, compared to traditional methods for calculating binding free energy.

### 2.4. Retrospective Application of FMOScore to Predict Binding Free Energy

**Figure 5:**
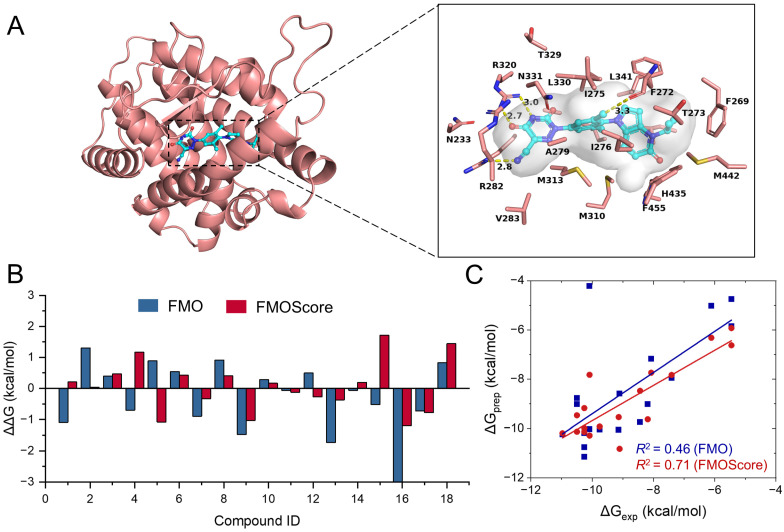
(**A**) Predicted binding pose of representative THR-β agonist (compound **12**) in human THR-β ligand-binding domain (PDB code: 1N46). Key residues are shown in stick. (**B**) Differences between FMOScore-based predictions, FMO-based predictions, and Δ*G*_exp_ for THR-β. (**C**) Correlations between experimentally measured affinities and Δ*G* values predicted by FMOScore (red dots) and FMO (blue square) for the THR-β agonists.

In the initial phase of our investigation, we conducted a retrospective evaluation of FMOScore’s performance concerning thyroid hormone receptor β (THR-β) agonists, using a set of 18 compounds taken from the work of Hu et al. [65] (Table S4 and Figure 5A). Overall, we observed that the FMOScore approach (*R*^2^ = 0.71) exhibited a better correlation with Δ*G*_exp_ compared to the FMO interaction energy (*R*^2^ = 0.46) (Figure 5C), which suggests that the solvation free energy and deformation energy contributions play a positive role in ligand binding. Table S4 reveals that the contributions of solvation free energy and deformation energy to the binding free energy are significant, with correlations *R*^2^ reaching 0.37 and 0.4, respectively. In detail, ligand **16** incorporated a strongly polar carboxyl moiety, which resulted in a notably elevated penalty in solvation free energy as computed using the COSMO solvent model. Ligands **3** to **5** exhibited a reduced number of flexible bonds, resulting in the lowest deformation energy penalties calculated from first principles (Table S4). This also underscored the rationality of the solvation free energy and deformation energy calculations established by this method. Moreover, the difference between Δ*G*_pred_ obtained by FMOScore and the experimental value remained within 2 kcal/mol, indicating its high prediction accuracy (Figure 5B). Hence, by means of solvation free energy and deformation energy calculations, the precision of FMO predictions for binding affinity can be further enhanced, thereby extending its applicability to a broader spectrum of drug design scenarios.

### 2.5. Design and Synthesis of Potent and Novel SHP-2 Inhibitors

**Scheme 1:**
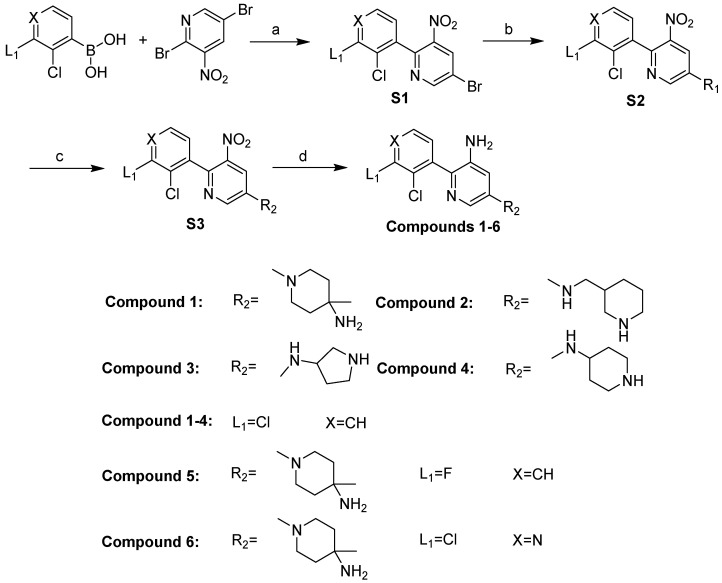
Synthesis route of compounds **1**~**6**. Reagents and conditions: (a) PdCl_2_(dppf), Cs_2_CO_3_, 1,4-dioxane, 80 °C, 25.7%; (b) XantPhos, Pd_2_(dba)_3_, Cs_2_CO_3_, 90 °C, 64.9%; (c) TFA, DCM, r.t., 81.7%; (d) Fe, CH_3_COOH/EtOH (1:1), 70 °C, 9.4%.

**Scheme 2:**
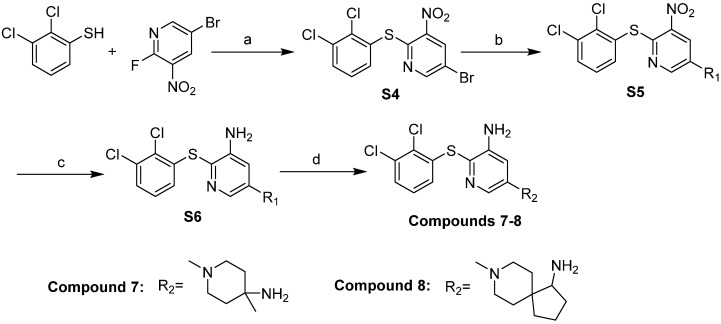
Synthesis route of compounds **7**~**8**. Reagents and conditions: (a) KOH, TBAF in THF, H_2_O, PhMe, r.t., 2 h, 51.7%; (b) XantPhos, Pd_2_(dba)_3_, Cs_2_CO_3_, 90 °C, 23.2%; (c) Fe, CH_3_COOH/EtOH (1:1), 70 °C, 63.7%; (d) TFA, DCM, r.t., 41.4%.

The synthesis of compound **1**–**8** and intermediate S1–S6 is shown in Scheme 1 and Scheme 2. Firstly, substituted phenylboronic acid and 2,5-dibromo-3-nitropyridine underwent a Suzuki coupling reaction catalyzed by palladium afforded intermediate S1. Then, S1 reacted with substituent amines to obtain intermediate S2. Subsequently, S2 was deprotected to remove the Boc group and then reduced by iron powder to obtain the final compounds **1**~**6** (Scheme 1). The initial step in the synthesis of compounds **7**–**8** involved a reaction between 3-dichlorophenol and 5-bromo-2-fluoro-3-nitropyridine, yielding intermediate S4. Then, S4 underwent the Suzuki coupling reaction with the corresponding substituted amine to obtain intermediate S5. Finally, S5 reduced the nitro group to an amino group by iron powder reduction, and then removed the Boc group to obtain the final compounds **7**~**8** (Scheme 2).

**Figure 6:**
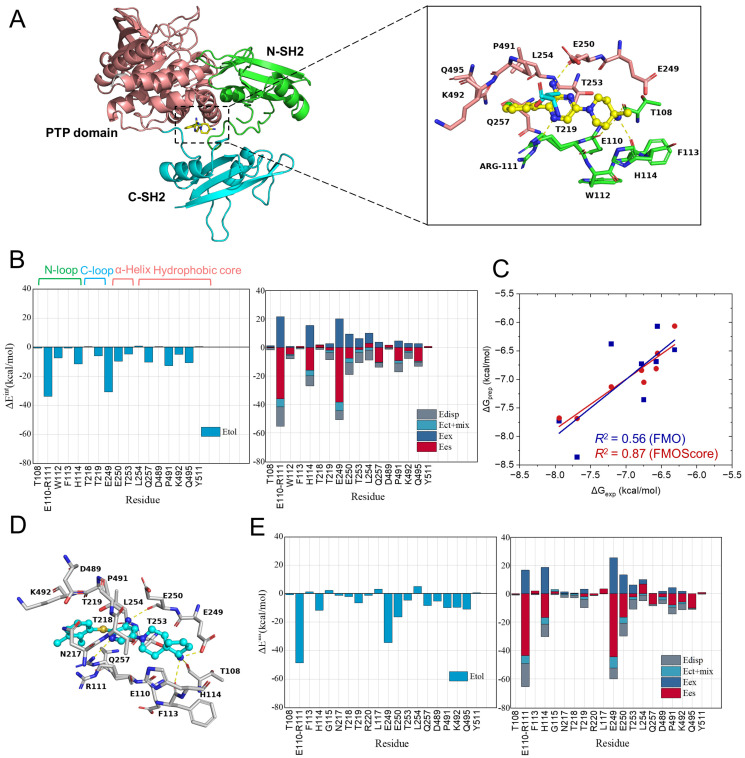
Discovery and design of novel SHP-2 inhibitors. (**A**) Binding mode of SHP-2 inhibitor **SHP099** (PDB code: 5EHR). Key residues are shown in stick. Hydrogen bonds are displayed as yellow dashes. (**B**) PIE between inhibitor **SHP099** and the residues at the allosteric site of SHP-2 (**left graph**), and PIEDA for these residues (**right graph**). The dispersion, exchange-repulsion, charge transfer, and electrostatic terms are represented in gray, cyan, blue, and red, respectively. (**C**) Correlations between experimentally measured affinities and Δ*G* values predicted by FMOScore for the designed SHP-2 inhibitors. (**D**) Predicted binding mode of compound **8**. (**E**) PIE and PIEDA for the residues at the allosteric site of SHP-2 and compound **8**.

It has been reported that **SHP099**, a lead compound, has an inhibitory role by locking SHP-2 in an auto-inhibited conformation, which binds to the central tunnel region formed at the C-SH2, N-SH2, and PTP domains (Figure 6A). The results of PIEDA on the X-ray crystal structure of SHP-2 (PDB: 5EHR) are shown in Figure 6B. The PIEDA results showed that the high electrostatic (ES) and charge transfer with inseparable (CT + MIX) terms of Arg111, followed by Glu250, indicate that these two residues formed a strong hydrogen bond with the ligand. In addition, the main PIEDA components of Glu249 (PTP) and His114 (N-SH2) were ES and CT + MIX terms. The former formed a salt bridge or ionic interaction with quaternary ammonium cation, and the latter formed a cation–π interaction with this cation group. Arg111 made a cation–π stacking interaction with the dichlorophenyl motif. The methyl group of Thr219 formed a CH–π interaction with pyrazine of **SHP099**, and its DI component was substantial at −5 kcal/mol. Moreover, five residues (Thr253, Leu254, Pro491, Gln257, and Lys492), whose main PIEDA component is dispersion interactions (DIs) (−3.0~−7.2 kcal/mol), were located around the dichlorophenyl group. This indicates that these residues form hydrophobic interactions with the ligand. The sum of PIE was equal to −141.7 kcal/mol (ES:EX:CT + MIX:DI = −131:98:−30:−77 kcal/mol), indicating a significant influence of electrostatic forces in the activity of this ligand. Given FMO analysis, the quaternary ammonium cation of SHP099 made apparent ionic interactions, which occupied a polar region of the allosteric binding pocket. The aminopyrazine ring acted as a H-bond acceptor and a H-bond donor to make key H-bonds with Arg111 and Glu250, respectively. Therefore, we applied the developed FMOScore method for rational drug design after analyzing the key pharmacophores of **SHP099**. 

The protein–ligand interactions of inhibitor **SHP099** with SHP-2 (PDB code 5EHR) were revealed by FMO analysis (Figure 6B). In attempts to build an SAR of the central ring for more novel compounds, we preserved the aniline interaction with Glu250 and replaced pyrazine with pyridine. The results showed that compound **1** retained biochemical inhibition (IC_50_ = 1.494 ± 0.024 μM, SHP2^PTP^ IC_50_ > 100 μM, Table 2 and Figure S4), which exhibited a 16-fold decrease compared to **SHP099** (IC_50_ = 0.092 ± 0.001 μM, SHP2^PTP^ IC_50_ > 100 μM). This is presumably due to changes in the binding affinity caused by differences in electron cloud density between pyrazine and pyridine.

In our attempt to improve the potency and obtain novel structures by modifying the piperazine motif, we extended the terminal amine toward a polar region for discovering new interactions. Morphing the piperazine ring to a piperidine-3-ylmethanamine motif and a piperidine-4-amine motif reduced inhibition 10-fold (compound **2**, IC_50_ = 15.610 ± 1.490 μM) and 15-fold (compound **4**, IC_50_ = 23.490 ± 2.860 μM), respectively. These compounds have low binding affinities predicted by FMOScore, which can be attributed to higher deformation energies resulting from an increased number of one or two rotation bonds (Table 2). Pyrrolidine-3-amine substituted for the piperazine ring (compound **3**, IC_50_ = 5.174 ± 1.080 μM) was approximately equipotent to compound **1** in biochemical assays, on account of it having less solvation free energy (Table 2). Further, our attention turned to the dichlorophenyl motif, which effectively filled a small hydrophobic pocket formed by Arg111, Thr253, Leu254, Gln257, and Gln495. Attempts to replace meta-chlorine with fluorine in the phenyl ring resulted in poor phosphatase inhibition (compound **5**, IC_50_ = 15.110 ± 0.800 μM). The redesign of the aryl group to include pyridyl N resulted in a more polar compound **6**, displaying reduced phosphatase inhibition (IC_50_ = 10.650 ± 0.920 μM) compared to compound **1**. These data indicated that the decrease in the hydrophobicity of compounds **5** and **6** affected the biochemical activity. Then, we introduced the thioether linker into the dichlorophenyl motif, which resulted in compound **7**. We evaluated compound **7** and found reduced biochemical potency (IC_50_ = 11.250 ± 1.190 μM) relative to compound **1**. In our attempt to improve the potency of SHP-2 biochemical inhibition, substituting the piperazine subunit in compound **7** with spirocyclic amine (compound **8**, IC_50_ = 2.290 ± 0.660 μM, Table 2 and Figure S4) found new interactions and increased the biochemical activity five-fold compared to that in compound **7**. FMOScore revealed that spirocyclic amine formed a CH–π interaction with His114 (Figure 6D,E), which occupied more ES components than compounds **7** (−16 kcal/mol vs. −5 kcal/mol). We observed a higher correlation (*R*^2^ = 0.87, Figure 6C) between the inhibitors’ biological potencies and the binding affinities calculated by FMOScore, as compared to the FMO method (*R*^2^ = 0.56). Despite the fact that SHP2 inhibitors consist of ionizable fragments, the incorporation of an implicit solvent model into the FMOScore method proved advantageous for assessing the accurate binding affinity. Those results suggested that the direction of structure optimization is reasonable for aminopyrazine core, dichlorophenyl, and piperazine motifs.

## 3. Materials and Methods

### 3.1. FMOScore Calculations

From a microscopic point of view, binding free energy (Δ*G*_bind_) is composed of the enthalpy change and entropy change (see also the thermodynamic cycle in Figure 1). In the drug discovery process, the half maximal inhibitory concentration (IC_50_) values are often experimentally measured for tested inhibitors. The dependence between IC_50_ or *K*_d_ and free energy of binding can be expressed [66] as follows:

(1) $$\Delta G_{\mathrm{bind}}=\Delta H-T\Delta S=-k_{B}T\log(K_{d})\approx -k_{B}T\log(IC_{50})$$

where *k_B_* and *T* stand for Boltzmann constant and temperature, respectively. 

The free energy of binding in solution, Δ*G*_bind_, is broken down into the gas-phase binding free energy (Δ$G_{\mathrm{bind}}^{\mathrm{gas}}$) and the solvation free energy (Δ*G*^sol^). The gas-phase free energy of binding contains an enthalpic term (Δ$H_{\mathrm{bind}}^{\mathrm{gas}}$) and an entropic term (*T*Δ$S_{\mathrm{bind}}^{\mathrm{gas}}$).

(2) $$\Delta G_{\mathrm{bind}}=\Delta G_{\mathrm{bind}}^{gas}+\Delta G^{sol}$$

(3) $$\Delta G_{\mathrm{bind}}^{gas}=\Delta H_{\mathrm{bind}}^{gas}-T\Delta S_{\mathrm{bind}}^{gas}$$

Δ$H_{\mathrm{bind}}^{\mathrm{gas}}$ is expressed as the difference of molecular energy in vacuum for the structures of the complex (“com”), receptor protein (“pro”), and isolated ligand (“lig”). *T*Δ$S_{\mathrm{bind}}^{\mathrm{gas}}$ is represented as the degrees of freedom of the individual systems, calculated by interaction entropy (IE) method.

(4) $$\Delta H_{\mathrm{bind}}^{gas}=E_{\mathrm{com}}-E_{\mathrm{pro}}-E_{\mathrm{lig}}$$

There is a difference between the energy of the isolated ligand conformation and the protein-bound ligand (“lig(com)”), due to fact that the ligand must take on strain in order to bind to the protein. Therefore, Δ$H_{\mathrm{bind}}^{\mathrm{gas}}$ is further separated into the intermolecular interaction energy (Δ*E*_int_), all in the bound state, and the deformation energy of the ligand (Δ$E_{\mathrm{lig}}^{\mathrm{def}}$).

(5) $$\Delta H_{\mathrm{bind}}^{gas}=\Delta E_{\mathrm{int}}+\Delta E_{\mathrm{lig}}^{\mathrm{def}}$$

(6) $$\Delta E_{\mathrm{int}}=E_{\mathrm{com}}-E_{\mathrm{pro}}-E_{\mathrm{lig}(\mathrm{com})}$$

(7) $$\Delta E_{\mathrm{lig}}^{\mathrm{def}}=E_{\mathrm{lig}(\mathrm{com})}-E_{\mathrm{lig}}$$

where Δ*E*_int_ is represented as the summation of IFIEs calculated by FMO calculations in vacuum (see below).

Finally, the solvation free energy upon complex formation is approximately divided into the change of solvation free energy in binding state (Δ*G*^sol^). Hence, Δ*G*_bind_ is directly estimated from FMOScore energies as follows:

(8) $$\Delta G_{\mathrm{bind}}=\Delta E_{\mathrm{int}}+\Delta E_{\mathrm{lig}}^{\mathrm{def}}-T\Delta S_{\mathrm{bind}}^{gas}+\Delta G^{sol}$$

Considering the entropy term is computationally expensive, the entropy term was dropped in FMOScore (detailed results can be found in Section 2.1).

**Structure preparation.** In this work, the structures of Schrödinger FEP+ benchmark and SHP-2–ligand complexes were obtained by crystal structures and molecular docking simulations. Residues within 4.5 Å surrounding the ligand were chosen as the entire system calculated. The missing hydrogen atoms of complexes were added at physiological pH (7.0), and the protonation states of the acidic and basic amino acid residues were predicted using the Protonate 3D module with the default settings (the generalized Born/volume integral methodology with a 15 Å cutoff and a solvent dielectric constant of 80). We used a constrained minimization procedure to refine the complexes with the semiempirical Amber10: EHT forced field implemented in Molecular Operating Environment (MOE version 2014.09) program, where each atom was allowed to deviate up to 0.5 Å from its original position in the crystal structure. The prepared complex structures were subjected to subsequent accurate FMO calculations. 

**Fragment molecular orbital (FMO) method and inter-fragment interaction energy (IFIE).** Here, the FMO method was applied to all systems using FMO code version 5.1 as embedded in General Atomic and Molecular Electronic Structure System (GAMESS), a general ab initio quantum chemistry package [14,67]. In this work, we used the second-order Møller−Plesset perturbation theory (MP2) [68] method with a 6-31G* basis set [69].

The FMO method is a general quantum mechanical method that divides large molecules, such as proteins, into *N* fragments. Each residue is characterized as a fragment, and the interaction energies reported herein correspond to essential amino acid residues, not fragments of residues. The total energy *E* of the whole system is calculated using the following series expansion (truncated here at the two-body terms, the FMO2 method [70]).

(9) $$E=\sum_{I}^{N}E_{I}+\sum_{I>J}^{N}\left(E_{IJ}-E_{I}-E_{J}\right)$$

where monomer (*I*) and dimer (*IJ*) energy are obtained as described above.

The value of IFIE as well as the pair interaction energy decomposition analysis (PIEDA) were used to investigate the chemical situations in pharmacophore. The fragment pair interaction energy (PIE) was obtained by subtracting the two monomer energies from the dimer energy. PIE does not indicate binding free energy, but rather the “strength” of the interaction between the ligand and the protein residues in the complex, which is enthalpy in gas phase. The IFIE between fragments *I* and *J* ($\Delta E_{IJ}^{\mathrm{int}}$) is decomposed into its energy components, such as electrostatic (ES), exchange (EX), charge transfer with inseparable (CT + MIX), and dispersion interactions (DI) [71], as follows:

(10) $$\Delta E_{IJ}^{\mathrm{int}}=\Delta E_{IJ}^{\mathrm{ES}}+\Delta E_{IJ}^{\mathrm{EX}}+\Delta E_{IJ}^{\mathrm{CT}+\mathrm{MIX}}+\Delta E_{IJ}^{\mathrm{DI}}$$

where the DI component corresponds to the MP2 electron correlation energy and the others constitute the Hartree−Fock (HF) energy.

**PM7/COSMO solvation.** For biological systems, a significant task to be described is the solvation phenomena of the complexes and the binding partners prior to binding, in addition to the complex interactions in vacuo. A compromise that balances speed and precision for the solvation is using the COSMO model employing the scaled-conductor approximation [72], which is implemented for PM7 calculations in MOPAC [73]. Kristiam et al.’s benchmark set showed that the PM7/COSMO solvation model is highly accurate in the calculation of solvation energy change upon binding [50]. 

**Deformation energy of ligands.** Ligands often distort the shape to adjust to the narrow space of the pocket at the deformation energy. The deformation energy of the ligand is obtained from the energy difference of single-point energy between the ligand-bound conformation and the low-energy conformation. For each molecule, 10,000 conformers were generated using Confab [74] as implemented in Open Babel. The generated conformers, clustered and deduplicated, were optimized using the GFN2-xTB method developed by Grimme [75,76]. The lowest energy conformers were then obtained by clustering, deduplicating, and sorting the conformations with Isostat program [77]. Moreover, the conformations with the top three lowest energies were optimized and computed the vibrational frequencies in the Gaussian09 (Revision C.01) [78] software package using the B3LYP method and the 6-31G(d,p) basis set in Molclus software [77]. Therefore, we obtained the lowest energy of molecules ab initio. Meanwhile, the single-point energy of ligand-bound conformation was calculated at the B3LYP/6–31G(d,p) level. In this method, it is recommended to add a dispersion correction, such as B3LYP-D3(BJ), to optimize the system when the small molecule exhibits significant intramolecular weak interactions [79]. In the presence of pronounced intramolecular weak interactions, particularly dispersion-dominated pi–pi stacking, neglecting dispersion correction may lead to substantial discrepancies between the optimized and actual structures [80].

**Interaction entropy calculation.** A novel entropy calculation method (named interaction entropy, IE) developed by Duan et al. [51] was used for the binding free energy calculation. Interaction entropy calculations are derived from protein–ligand interactions in the gas phase, obtained from MD simulation, and represent fluctuations in ligand–receptor interactions (the derivation process can be found in reference [51]). A 2 ns MD simulation was previously performed for each system at a constant temperature (300 K). Furthermore, 100,000 snapshots were extracted from the last 1 ns trajectories for interaction entropy calculations. Equation (11) shows the components for calculating IE, where $\Delta E_{\mathrm{pl}}^{\mathrm{int}}=E_{\mathrm{pl}}^{\mathrm{int}}-\langle E_{\mathrm{pl}}^{\mathrm{int}}\rangle$ represents the fluctuation of protein–ligand interaction energy around mean energy.

(11) $$-T\Delta S=KT\ln\langle e^{\beta \Delta E_{\mathrm{pl}}^{\mathrm{int}}}\rangle$$

where 〈〉 represents the ensemble average. *β*, *K*, and *T* are the inverse of the temperature (1/*kT*), Boltzmann constant, and simulation temperature (300 K), respectively.

### 3.2. Comparison with Traditional Methods

The relative binding free energy data were obtained from the publicly available Schrödinger FEP+ benchmark data set [48]. The MM/PB(GB)SA calculations were performed to obtain the binding energy of the complex implemented in mmpbsa.py tool of Amber18 [81]. Snapshot conformations were extracted from the short-time MD simulation every 10 ps, from 1 ns to 2 ns, for a total of 101 snapshots. The binding conformation of proteins and ligands was scored by Autodock vina [82] with default settings.

### 3.3. Statistical Analyses

Correlation statistics and error estimation were evaluated with the bootstrap method with 10,000 bootstrap repetitions using standard Python 3 with NumPy and SciPy libraries [83]. 

### 3.4. Protein Expression, Purification, and Biochemical Assay

SHP2^WT^ (1–593) and SHP2^PTP^ (237–529) genes were inserted separately into a pET30 vector, and SHP2 recombinant proteins were expressed and purified according to protocols published previously [84]. Briefly, the constructs were transformed into BL21 Star^TM^ (DE3) cells and cultured at 37 °C for 3~4 h. Subsequently, the cells with high expression of SHP2 protein were harvested after being induced by IPTG and grown overnight at 18 °C. Cell pellets were resuspended in lysis buffer (25 mM imidazole, 50 mM Tris−HCl, 500 mM NaCl, 2.5 mM MgCl_2_, 1 mM tris(2-carboxyethyl)phosphine (TCEP), 1 μg/mL DNase1, and complete EDTA-free protease inhibitor) and lysed using Low Temperature Ultrahigh Pressure Continuous Flow Cell Disrupter (JNBIO), followed by ultracentrifugation. The supernatant was collected. Then, the SHP2 was purified from the collected supernatant by using HisTrap HP chelation column, TEV protease, and HiTrap Q FastFlow column. 

To access the catalytic activity of SHP-2, a prompt fluorescence assay was adopted, which was conducted in a 384-well black polystyrene plate (Perkin Elmer, cat. no. 6008260). We performed the phosphatase reactions in 384-well black polystyrene plates with a final reaction volume of 20 μL, which consisted of 12.5 μL of assay buffer (75 mM NaCl, 75 mM KCl, 60 mM HEPES, pH 7.2, 1 mM EDTA, 0.05% P-20, 5 mM DTT), 2.5 μL of peptide IRS1_pY1172 (dPEG8) pY1222 (Shanghai Shengzhao Biotech Co., Ltd. cat. no. 79319-2, Shanghai, China), 2.5 μL of test compounds, and 2.5 μL of SHP2^WT^ or SHP2^PTP^. Then, the mixture was then incubated at 25 °C for 60 min. To initiate the reaction, the surrogate substrate DiFMUP (Invitrogen, cat. no. D6567, Shuzhou, China, 200 μM) was added and incubated at 25 °C for another 30 min. Finally, 5 μL of a 160 μM bpV(Phen) (Shanghai Hongye Biotechnology Co., Ltd., cat. no. sc-221378, Shanghai, China) was added to terminate the reaction. Fluorescence signal was detected using a microplate reader (Bio-Tek, Shanghai, China, Synergy2) at Ex/Em = 340/450 nm. Using control-based normalization, normalized IC_50_ regression curves were fitted to inhibitor dose response curves. The lead compound **SHP099** (Guangzhou Qiyun Biotechnology Co., Ltd., cat. no. 1801747-42-1, Guangzhou, China) was measured as the positive control, and at least three parallels were set for each experiment. 

### 3.5. Chemistry

**General Methods.** All the compounds were synthesized through the routes depicted in Scheme 1 and Scheme 2. **S1** to **S6** were intermediate compounds, and compounds **1** to **10** were the final designed compounds. The reagents and solvents used in the chemical experiment were purchased from commercial sources, and could be used directly without further purification. Column chromatography silica gel chromatography (300~400 mesh) was purchased from Shanghai Titan Technology Co., Ltd., produced by Qingdao Marine Chemical Factory. Reaction progress was monitored using thin layer chromatography (TLC) and a WFH-204B UV analyzer was used for thin layer chromatography. ^1^H NMR and ^13^C NMR data were collected by Brucker AM-400 (400 MHz) or Ascend 600 (600 MHz) NMR instruments with chemical shifts expressed in ppm (deuterated reagents were CDCl_3_ or DMSO-d_6_ and used TMS as the internal standard). High-resolution mass spectrometry HRMS data were completed by the Analytical Test Center of East China University of Science and Technology (Xevo G2 TOF MS). The purity of all final products was determined by our laboratory’s High Performance Liquid Chromatograph System instrument (with the Agilent TC-18, 5 µm, 4.6 mm × 250 mm reversed-phase column) and the purity of all compounds was above 95%. The apparatus for testing the melting point was a WRS-1B Digital Melting Point apparatus (WRS-1B).

**S1: 5-bromo-2-(2,3-dichlorophenyl)-3-nitropyridine.** 2,3-dichlorophenylboric acid (3.00 g, 15.70 mmol), 2,5-dibromo-3-nitropyridine (4.40 g, 15.70 mmol), PdCl_2_(dppf), and Cs_2_CO_3_ were dissolved in dry 1,4-dioxane under N_2_ atmosphere. The mixture was reacted at 90 °C for 2 h. After completion of the reaction, the solution was filtered through celite and the filtrate was extracted with EA, concentrated, and purified by column chromatography to obtain **S1** (1.40 g, 25.7%) as a yellow solid. ^1^H NMR (400 MHz, DMSO-*d*_6_) δ 9.20 (d, *J* = 2.0 Hz, 1H), 8.98 (d, *J* = 2.0 Hz, 1H), 7.79 (dd, *J* = 6.4, 3.2 Hz, 1H), 7.52 (d, *J* = 3.2 Hz, 1H), 7.51 (t, 1H). LC-MS (ESI) calculated for C_11_H_5_BrCl_2_N_2_O_2_ [M + H]^+^ 346.89, found 347.05.

**S2: tert-butyl (1-(6-(2,3-dichlorophenyl)-5-nitropyridin-3-yl)-4-methylpiperidin-4-yl) carbamate.** 5-bromo-2-(2,3-dichlorophenyl)-3-nitropyridine (1) (0.50 g, 1.44 mmol), tert-butyl (4-methylpiperidin-4-yl) carbamate (0.34 g, 1.59 mmol), Cs_2_CO_3_ (1.40 g, 4.30 mmol), Xantphos (0.17 g, 0.29 mmol), and Pd_2_(dba)_3_ (0.14 g, 0.15 mmol) were dissolved in dry DMF under N_2_ atmosphere. The mixture was reacted at 90 °C for 1 h. After completion of the reaction, the solution was filtered through celite and the filtrate was extracted with EA, concentrated, and purified by column chromatography to obtain **S2** (0.45 g, 64.9%) as a yellow solid. ^1^H NMR (400 MHz, DMSO-*d*_6_) δ 8.67 (d, *J* = 2.7 Hz, 1H), 7.91 (d, *J* = 2.6 Hz, 1H), 7.71 (dd, *J* = 7.8, 1.7 Hz, 1H), 7.47 (t, *J* = 7.8 Hz, 1H), 7.42 (dd, *J* = 7.7, 1.7 Hz, 1H), 6.66 (s, 1H), 3.66–3.58 (m, 2H), 3.20 (t, *J* = 10.7 Hz, 2H), 2.16 (m, *J* = 12.5 Hz, 2H), 1.59–1.51 (m, 2H), 1.39 (s, 9H), 1.27 (s, 3H). LC-MS (ESI) calculated for C_22_H_26_Cl_2_N_4_O_4_ [M + H]^+^ 481.13, found 481.20.

**S3: 1-(6-(2,3-dichlorophenyl)-5-nitropyridin-3-yl)-4-methylpiperidin-4-amine.** Tertbutyl (1-(6-(2,3-dichlorophenyl)-5-nitropyridin-3-yl)-4-methylpiperidin-4-yl) carbamate (2) (0.45 g, 0.93 mmol) in DCM (4 mL) was added to TFA (1 mL). The mixture was reacted at room temperature for 10 min. After completion of the reaction, the solution was quenched with saturated aqueous Na_2_CO_3_ extracted with DCM, dried (Na_2_SO_4_), and concentrated under reduced pressure to give **S3** (0.29 g, 81.7%) as a yellow solid. LC-MS (ESI) calculated for C_17_H_18_Cl_2_N_4_O_2_ [M + H]^+^ 381.08, found 381.15.

**Compound 1: 5-(4-amino-4-methylpiperidin-1-yl)-2-(2,3-dichlorophenyl) pyridin-3-amine.** Fe (0.21 g, 3.75 mmol) was added to the DCM solution of 1-(6-(2,3-dichlorophenyl)-5-nitropyridin-3-yl)-4-methylpiperidin-4-amine (0.29 g, 0.76 mmol) and the mixed solution of CH_3_COOH/CH_3_CH_2_OH (1:1). The mixture was reacted at 70 °C for 1 h. After completion of the reaction, the solution was quenched with saturated aqueous Na_2_CO_3_. Then, the solution was filtered through celite and the filtrate was extracted with EA, dried and concentrated, and then purified by column chromatography to obtain compound **1** (0.025 g, 9.4%) as a yellow solid. Mp: 170.5–171.2 °C. ^1^H NMR (400 MHz, DMSO-*d*_6_) δ 7.63 (d, *J* = 1.8 Hz, 1H), 7.61 (d, *J* = 1.5 Hz, 1H), 7.39 (t, *J* = 7.8 Hz, 1H), 7.28 (dd, *J* = 7.6, 1.5 Hz, 1H), 6.63 (d, *J* = 2.4 Hz, 1H), 4.71 (s, 2H), 3.25–3.19 (m, 4H), 1.51 (m, *J* = 19.6, 11.3, 6.5 Hz, 4H), 1.10 (s, 3H). ^13^C NMR (151 MHz, DMSO-*d*_6_) δ 147.70, 142.39, 140.86, 132.62, 132.43, 131.89, 131.04, 129.98, 128.61, 126.76, 107.27, 47.33, 44.67, 38.92, 30.15. HRMS (ESI): (*m*/*z*): (M + H)^+^ calculated for C_17_H_20_Cl_2_N_4_, 351.1065; found 351.1141. HPLC purity: 99.7%, retention time = 6.39 min.

**Compound 2: 2-(2,3-dichlorophenyl)-N5-(piperidin-3-ylmethyl) pyridine-3,5-diamine.** Prepared following the same procedure as compound 1 with a yield of 15.3%. Mp: 182.2–183.6 °C. ^1^H NMR (600 MHz, DMSO-*d*_6_) δ 7.58 (dd, *J* = 8.0, 1.2 Hz, 1H), 7.36 (t, *J* = 7.8 Hz, 1H), 7.32 (d, *J* = 2.2 Hz, 1H), 7.26 (dd, *J* = 7.6, 1.2 Hz, 1H), 6.24 (d, *J* = 2.1 Hz, 1H), 5.76 (t, *J* = 5.5 Hz, 1H), 4.59 (s, 2H), 3.02 (d, *J* = 10.2 Hz, 1H), 2.84–2.81 (m, 2H), 2.44 (dd, *J* = 16.0, 6.8 Hz, 1H), 2.21 (t, *J* = 10.8 Hz, 1H), 1.81 (d, *J* = 10.8 Hz, 1H), 1.71–1.63 (m, 1H), 1.57 (dd, *J* = 9.5, 3.5 Hz, 1H), 1.35 (dd, *J* = 23.9, 11.9 Hz, 1H), 1.11–1.03 (m, 1H). ^13^C NMR (151 MHz, DMSO-*d*_6_) δ 146.05, 142.52, 141.21, 132.36, 132.02, 131.20, 129.70, 128.49, 125.02, 102.80, 47.04, 46.69, 36.07, 29.43, 25.69. HRMS (ESI): (*m*/*z*): (M + H)^+^ calculated for C_17_H_20_Cl_2_N_4_, 351.1065; found 351.1142. HPLC purity: 95.7%, retention time = 4.11 min.

**Compound 3: 2-(2,3-dichlorophenyl)-N5-(pyrrolidin-3-yl) pyridine-3,5-diamine.** Prepared following the same procedure as compound 1 with a yield of 23.1%. Mp: 184.7–185.1 °C. ^1^H NMR (600 MHz, DMSO-*d*_6_) δ 7.59 (d, *J* = 7.6 Hz, 1H), 7.37 (t, *J* = 7.8 Hz, 1H), 7.31 (s, 1H), 7.26 (d, *J* = 7.3 Hz, 1H), 6.27 (s, 1H), 5.75 (d, *J* = 6.4 Hz, 1H), 4.62 (s, 2H), 3.71 (d, *J* = 2.4 Hz, 1H), 2.99 (dd, *J* = 10.9, 6.1 Hz, 1H), 2.87 (dt, *J* = 14.5, 7.4 Hz, 1H), 2.75 (dd, *J* = 14.9, 9.2 Hz, 1H), 2.61 (dd, *J* = 11.0, 3.6 Hz, 1H), 2.03–1.93 (m, 1H), 1.55 (d, *J* = 4.9 Hz, 1H), 1.24 (d, *J* = 14.6 Hz, 1H). ^13^C NMR (151 MHz, DMSO-*d*_6_) δ 145.40, 142.49, 141.18, 132.37, 132.01, 131.19, 130.77, 129.73, 128.50, 125.36, 103.53, 53.74, 53.69, 45.94, 33.61. HRMS (ESI): (*m*/*z*): (M + H)^+^ calculated for C_15_H_16_Cl_2_N_4_, 323.0752; found 323.0832. HPLC purity: 96.2%, retention time = 4.36 min.

**Compound 4: 2-(2,3-dichlorophenyl)-N5-(piperidin-4-yl) pyridine-3,5-diamine.** Prepared following the same procedure as compound 1 with a yield of 18.5%. Mp: 169.9–171.7 °C. ^1^H NMR (400 MHz, DMSO-*d*_6_) δ 7.58 (dd, *J* = 8.0, 1.4 Hz, 1H), 7.36 (t, *J* = 7.8 Hz, 1H), 7.31 (d, *J* = 2.2 Hz, 1H), 7.25 (dd, *J* = 7.6, 1.4 Hz, 1H), 6.27 (d, *J* = 2.2 Hz, 1H), 5.58 (d, *J* = 7.8 Hz, 1H), 4.58 (s, 2H), 3.20–3.09 (m, 1H), 2.94 (d, *J* = 12.5 Hz, 2H), 2.53 (s, 1H), 2.48 (s, 1H), 1.85 (d, *J* = 10.8 Hz, 2H), 1.25–1.19 (m, 2H), 1.17 (t, *J* = 7.1 Hz, 1H). ^13^C NMR (151 MHz, DMSO-*d*_6_) δ 144.84, 142.55, 141.20, 132.37, 132.01, 131.20, 130.53, 129.69, 128.48, 125.43, 103.20, 50.02, 45.55, 33.71. HRMS (ESI): (*m*/*z*): (M + H)^+^ calculated for C_16_H_18_Cl_2_N_4_, 337.0909; found 337.0988. HPLC purity: 96.2%, retention time = 4.13 min.

**Compound 5: 5-(4-amino-4-methylpiperidin-1-yl)-2-(2-chloro-3-fluorophenyl) pyridin-3-amine.** Prepared following the same procedure as compound 1 with a yield of 25.9%. Mp: 173.5–174.3 °C. ^1^H NMR (400 MHz, DMSO-*d*_6_) δ 7.64 (d, *J* = 2.5 Hz, 1H), 7.44–7.36 (m, 2H), 7.19–7.14 (m, 1H), 6.63 (d, *J* = 2.5 Hz, 1H), 4.71 (s, 2H), 3.21 (d, *J* = 5.1 Hz, 4H), 1.54–1.44 (m, 4H), 1.08 (s, 3H). ^13^C NMR (151 MHz, DMSO-*d*_6_) δ 156.36, 146.68, 141.50, 139.72, 130.42, 127.62, 127.57, 126.81, 125.78, 119.41, 119.30, 114.88, 114.74, 106.12, 45.89, 43.62, 29.61. HRMS (ESI): (*m*/*z*): (M + H)^+^ calculated for C_17_H_20_ClFN_4_, 335.1361; found 335.1437. HPLC purity: 98.6%, retention time = 5.14 min.

**Compound 6: 5-(4-amino-4-methylpiperidin-1-yl)-2′,3′-dichloro-[2,4′-bipyridin]-3-amine.** Prepared following the same procedure as compound 1 with a yield of 22.5%. Mp: 189.6–191.3 °C. ^1^H NMR (400 MHz, DMSO-*d*_6_) δ 8.35 (d, *J* = 4.9 Hz, 1H), 7.66 (d, *J* = 2.4 Hz, 1H), 7.38 (d, *J* = 4.9 Hz, 1H), 6.59 (d, *J* = 2.4 Hz, 1H), 5.00 (s, 2H), 3.26–3.18 (m, 4H), 1.44 (dd, *J* = 12.0, 7.2 Hz, 4H), 1.07 (s, 3H). ^13^C NMR (151 MHz, DMSO-*d*_6_) δ 148.51, 148.19, 147.06, 146.42, 141.68, 128.51, 128.32, 125.93, 125.48, 105.74, 45.91, 43.39, 38.03, 29.67. HRMS (ESI): (*m*/*z*): (M + H)^+^ calculated for C_16_H_19_Cl_2_N_5_, 352.1018; found 352.1095. HPLC purity: 99.2%, retention time = 5.01 min.

**S4: 5-bromo-2-((2,3-dichlorophenyl) thio)-3-nitropyridine.** The solution of 2,3-dichlorophenol (1.00 g, 5.60 mmol), 5-bromo-2-fluoro-3-nitropyridine (1.24 g, 5.60 mmol), and KOH (0.47 g, 8.40 mmol) in PhMe was added dropwise of TBAF in THF and water. The mixture was reacted at room temperature for 2 h. After completion of the reaction, the solution was extracted with EA, concentrated, and purified by column chromatography to obtain **S4** (1.11 g, 51.7%) as a yellow solid. ^1^H NMR (600 MHz, DMSO-*d*_6_) δ 8.89 (d, *J* = 2.1 Hz, 1H), 8.82 (d, *J* = 2.1 Hz, 1H), 7.81 (dd, *J* = 8.1, 1.2 Hz, 1H), 7.74 (dd, *J* = 7.7, 1.4 Hz, 1H), 7.47 (t, *J* = 7.9 Hz, 1H). LC-MS (ESI) calculated for C_11_H_5_BrCl_2_N_2_O_2_S [M + H]^+^ 378.86, found 379.10.

**S5: Tertbutyl(1-(6-((2,3-dichlorophenyl)thio)-5-nitropyridin-3-yl)-4-methylpiperidin-4-yl) carbamate.** 5-bromo-2-((2,3-dichlorophenyl) thio)-3-nitropyridine (compound 4) (0.50 g, 1.30 mmol), tert-butyl (4-methylpiperidin-4-yl)carbamate (0.31 g, 1.40 mmol), Cs_2_CO_3_ (0.86 g, 2.60 mmol), Pd_2_(dba)_3_ (0.12 g, 0.13 mmol), and XantPhos (0.15 g, 0.26 mmol) were dissolved in dry DMF under N_2_ atmosphere. The mixture was reacted at 90 °C for 1 h. After completion of the reaction, the solution was filtered through celite and the filtrate was extracted with EA, concentrated, and purified by column chromatography to obtain **S5** (0.30 g, 23.3%) as a yellow solid. ^1^H NMR (400 MHz, DMSO-*d*_6_) δ 8.47 (d, *J* = 2.0 Hz, 1H), 8.40 (d, *J* = 2.1 Hz, 1H), 7.46 (d, *J* = 7.2 Hz, 1H), 7.26 (t, *J* = 8.0 Hz, 1H), 6.84–6.80 (m, 1H), 6.74 (s, 1H), 3.53 (d, *J* = 13.7 Hz, 2H), 3.27 (d, *J* = 11.1 Hz, 2H), 2.13 (d, *J* = 12.8 Hz, 2H), 1.50 (t, *J* = 10.3 Hz, 2H), 1.40 (s, 9H), 1.26 (s, 3H). LC-MS (ESI) calculated for C_22_H_26_Cl_2_N_4_O_4_S [M + H]^+^ 513.11, found 513.15.

**S6: tert-butyl(1-(5-amino-6-((2,3-dichlorophenyl)thio)pyridin-3-yl)-4-methylpiperidin-4yl)carbamate.** Fe (0.16 g, 2.90 mmol) was added to the dichloromethane solution of tert-butyl (1-(6-((2,3-dichlorophenyl) thio)-5-nitropyridin-3-yl)-4-methylpiperidin-4-yl) carbamate (0.30 g, 0.58 mmol) and mixed solution of CH_3_COOH/CH_3_CH_2_OH (1:1). The mixture was reacted at 70 °C for 1 h. After completion of the reaction, the solution was quenched with saturated aqueous Na_2_CO_3_. Then, the solution was filtered through celite and the filtrate was extracted with EA, concentrated, and purified by column chromatography to obtain **S6** (0.12 g, 63.7%) as a yellow solid. ^1^H NMR (400 MHz, DMSO-*d*_6_) δ 7.68 (d, *J* = 2.1 Hz, 1H), 7.43 (dd, *J* = 8.0, 1.3 Hz, 1H), 7.25 (t, *J* = 8.0 Hz, 1H), 7.04 (d, *J* = 2.1 Hz, 1H), 6.69 (dd, *J* = 8.1, 1.3 Hz, 1H), 6.49 (s, 1H), 5.04 (s, 2H), 3.18–3.08 (m, 2H), 2.93 (t, *J* = 10.8 Hz, 2H), 2.11 (d, *J* = 12.1 Hz, 2H), 1.63 (dd, *J* = 16.8, 6.7 Hz, 2H), 1.39 (s, 9H), 1.27 (s, 3H). LC-MS (ESI) calculated for C_22_H_28_Cl_2_N_4_O_2_S [M + H]^+^ 483.13, found 483.15.

**Compound 7: 5-(4-amino-4-methylpiperidin-1-yl)-2-((2,3-dichlorophenyl)thio)pyridin-3-amine.** To a solution of tert-butyl (1-(5-amino-6-((2,3-dichlorophenyl)thio)pyridin-3-yl)-4-methylpiperidin-4-yl)carbamate (0.12 g, 0.31 mmol) in DCM was added dropwise a 3.0 M solution of TFA (0.5 mL, 1.5 mmol). The mixture was reacted at room temperature for 20 min. After completion of the reaction, the solution was quenched with saturated aqueous Na_2_CO_3_ extracted with DCM, dried (Na_2_SO_4_), and concentrated under reduced pressure to give **7** (0.02 g, 41.4%) as a white solid. Mp: 149.7–151.9 °C. ^1^H NMR (400 MHz, CDCl_3_) δ 7.89 (d, 1H), 7.21 (d, *J* = 7.8 Hz, 1H), 7.03 (d, 1H), 6.99 (t, *J* = 8.0 Hz, 1H), 6.69 (d, *J* = 7.9 Hz, 1H), 3.82 (s, 2H), 3.22–3.11 (m, 4H), 1.72 (d, *J* = 7.1 Hz, 4H), 1.64 (s, 2H), 1.23 (s, 3H). ^13^C NMR (151 MHz, CDCl_3_) δ 152.52, 143.02, 140.55, 136.03, 133.41, 129.13, 127.28, 126.91, 126.85, 125.73, 121.57, 47.41, 45.41, 40.29. HRMS (ESI): (*m*/*z*): (M + H)^+^ calculated for C_17_H_20_Cl_2_N_4_S, 383.0786; found 383.0863. HPLC purity: 95.1%, retention time = 5.47 min.

**Compound 8: 8-(5-amino-6-((2,3-dichlorophenyl)thio)pyridin-3-yl)-8-azaspiro[4.5]decan-1-amine.** Prepared following the same procedure as compound 7 with an 18.4% yield. Mp: 179.5–180.5 °C. ^1^H NMR (600 MHz, CDCl_3_) δ 7.90 (d, *J* = 2.0 Hz, 1H), 7.21 (dd, *J* = 8.0, 1.3 Hz, 1H), 7.03 (d, *J* = 2.0 Hz, 1H), 7.00 (t, *J* = 8.0 Hz, 1H), 6.69 (dd, *J* = 8.0, 1.2 Hz, 1H), 3.84 (s, 2H), 3.38 (dd, *J* = 16.3, 12.5 Hz, 2H), 2.93 (m, *J* = 19.1, 12.1, 2.4 Hz, 2H), 2.86 (t, *J* = 7.4 Hz, 1H), 2.01 (m, *J* = 11.5, 6.0, 3.5 Hz, 1H), 1.84 (m, *J* = 13.1, 9.3, 5.6 Hz, 1H), 1.75 (dd, *J* = 12.5, 4.0 Hz, 1H), 1.71 (dd, *J* = 12.5, 3.8 Hz, 2H), 1.66–1.57 (m, 2H), 1.41 (t, *J* = 9.6 Hz, 1H), 1.37 (m, *J* = 11.4, 7.6, 1.7 Hz, 2H). ^13^C NMR (151 MHz, CDCl_3_) δ 152.87, 143.07, 140.64, 136.15, 133.39, 129.04, 127.27, 126.86, 126.77, 125.66, 121.36, 61.44, 46.53, 45.82, 43.23, 36.07, 33.09, 29.21, 19.80. HRMS (ESI): (*m*/*z*): (M + H)^+^ calculated for C_20_H_24_Cl_2_N_4_S, 423.1099; found 423.1179. HPLC purity: 97.2%, retention time = 8.17 min.

## 4. Conclusions

Quantum mechanics (QM) methods are increasingly used in drug discovery and are rapidly replacing classical molecular mechanics (MM) methods to elucidate the full complexity of molecular interactions. In this study, we introduced FMOScore, a novel QM-based method for calculating binding free energy. FMOScore employs linear fitting on energy terms including gas-phase potential energy, deformation energy, and solvation free energy. Evaluating FMOScore’s performance on the Schrödinger FEP+ benchmark reveals good correlation (average Pearson *R* = 0.$87_{0.89}^{0.84}$, average Kendall *τ* = 0.$69_{0.72}^{0.65}$) and higher prediction accuracy compared to other widely used traditional methods. The results obtained from retrospective analyses showed that the calculation of solvation free energy and deformation energy further enhanced the accuracy of FMO predictions for binding affinity, increasing the *R*^2^ from 0.46 to 0.71. Furthermore, utilizing FMOScore for structural optimization targeting SHP-2 led to the discovery of a novel and potent allosteric inhibitor (compound **8**), illustrating the effectiveness of FMOScore in practical structure-based drug design projects. FMOScore could be promising to provide reliable data for physics-informed deep learning models in drug–target interaction (DTI) predictions, further improving the model generalization.

## Figures and Tables

**Table 1:** Performance comparison of FMOScore with different function forms in Schrödinger FEP+ benchmark.

No.	Linear Fit Form	Performance Evaluation Metrics				
		*R* ^a^	*ρ* ^a^	*τ* ^a^	MUE ↓	RMSE
1	FMO	0.$62_{0.68}^{0.55}$ (0.0%)	0.$64_{0.70}^{0.57}$ (0.0%)	0.$47_{0.52}^{0.41}$ (0.0%)	0.$91_{1.00}^{0.81}$ (0.0%)	1.$14_{1.26}^{1.02}$ (0.0%)
2	FMO_PBSA	0.$81_{0.84}^{0.78}$ (30.6%↑)	0.$81_{0.84}^{0.77}$ (26.5%↑)	0.$61_{0.64}^{0.57}$ (29.7%↑)	0.$64_{0.71}^{0.57}$ (−29.6%↓)	0.$83_{0.92}^{0.74}$ (−27.1%↓)
3	FMO_GBSA	0.$82_{0.85}^{0.79}$ (32.2%↑)	0.$81_{0.84}^{0.77}$ (26.5%↑)	0.$62_{0.65}^{0.58}$ (31.9%↑)	0.$63_{0.70}^{0.57}$ (−30.7%↓)	0.$81_{0.91}^{0.72}$ (−28.9%↓)
4	FMO_COSMO	0.$85_{0.88}^{0.82}$ (37.1%↑)	0.$86_{0.88}^{0.83}$ (34.3%↑)	0.$67_{0.70}^{0.64}$ (42.5%↑)	0.$56_{0.62}^{0.49}$ (−38.4%↓)	0.$74_{0.83}^{0.65}$ (−35.0%↓)
5	FMO_PCM	0.$43_{0.60}^{0.26}$ (−30.6%↓)	0.$68_{0.75}^{0.61}$ (6.2%↑)	0.$51_{0.56}^{0.45}$ (8.5%↑)	1.$02_{1.14}^{0.91}$ (12.0%↑)	1.$36_{1.71}^{1.15}$ (19.2%↑)
6	FMO_SMD	0.$73_{0.77}^{0.68}$ (17.7%↑)	0.$75_{0.80}^{0.69}$ (17.1%↑)	0.$56_{0.60}^{0.51}$ (19.1%↑)	0.$77_{0.85}^{0.68}$ (−15.3%↓)	0.$99_{1.10}^{0.88}$ (−13.1%↓)
7	FMO_COSMO_SE	0.$87_{0.89}^{0.84}$ (40.3%↑)	0.$87_{0.89}^{0.84}$ (35.9%↑)	0.$69_{0.72}^{0.65}$ (46.8%↑)	0.$53_{0.60}^{0.47}$ (−41.7%↓)	0.$71_{0.80}^{0.62}$ (−37.7%↓)
8	FMO_COSMO_SE_IE	0.$87_{0.89}^{0.84}$ (40.3%↑)	0.$87_{0.89}^{0.84}$ (35.9%↑)	0.$68_{0.72}^{0.65}$ (44.6%↑)	0.$53_{0.60}^{0.47}$ (−41.7%↓)	0.$71_{0.80}^{0.62}$ (−37.7%↓)

^a^ *R*, *ρ*, *τ* are the Pearson, Spearman, Kendall correlation coefficients between Δ*G*_exp_ and the calculated scores using a publicly available Schrödinger FEP+ benchmark of 8 targets containing 258 complexes, respectively. MUE is the mean unsigned error. RMSE is the root-mean-squared error between Δ*G*_exp_ and scores. For each performance metric, 90% symmetric confidence intervals were estimated via bootstrap sampling with 10,000 resampling events and are reported as $X_{X_{low}}^{X_{high}}$, where *X* is the mean statistic and *X_low_* and *X_high_* are the values of the statistic at the 5th and 95th percentiles of the value-sorted list of the bootstrap samples. The arrows indicate whether Functions 2~8 show increases or decreases in five evaluation criteria in comparison to Function 1 (FMO method). The percentage values in brackets indicate the relative change of metrics computed by other functions in comparison to those calculated by Function 1 (FMO method).

**Table 2:** Structure–activity relationship study of SHP-2 inhibition activities.

			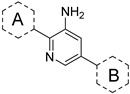					
Compound	Part A	Part B	SHP-2 IC_50_(μM) ^a^	Δ*G*_exp_ ^b^	FMOScore (kcal/mol)			
					Δ*G*_pred_ ^c^	Δ*E*^int^	Δ$E_{\mathrm{lig}}^{\mathrm{def}}$	Δ*G*^sol^
**SHP099**	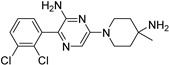		0.092 ± 0.001	−9.593	−7.964	−141.73	7.55	196.22
**1**	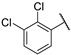	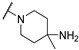	1.494 ± 0.024	−7.943	−7.677	−146.17	9.58	190.18
**2**	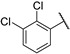	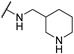	15.610 ± 1.490	−6.553	−6.544	−111.51	9.72	189.51
**3**	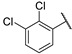	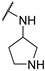	5.174 ± 1.080	−7.207	−7.129	−118.01	7.74	190.95
**4**	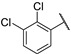	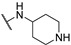	23.490 ± 2.860	−6.311	−6.066	−120.11	13.06	173.71
**5**	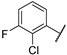	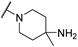	15.110 ± 0.800	−6.573	−6.812	−124.42	9.76	173.78
**6**	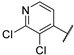	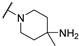	10.650 ± 0.920	−6.780	−6.840	−125.27	10.25	184.50
**7**	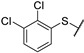	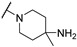	11.250 ± 1.190	−6.747	−7.049	−138.37	11.70	191.28
**8**	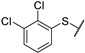	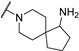	2.290 ± 0.660	−7.690	−7.685	−159.38	12.18	198.02

^a^ All the data are averages of three independent experiments (see the Section 3 for details). ^b^ Experimental affinities were converted to Δ*G*_exp_ values using the equation Δ*G*_exp_ ≈ *k_B_T*log IC_50._^c^ Predicted binding free energy Δ*G*_prep_ was fitted to the three types of energies calculated by FMOScore, consisting of binding affinities in vacuo (Δ*E*^int^), solvation free energy (Δ*G*^sol^), and deformation energy into the isolated form (Δ$E_{\mathrm{lig}}^{\mathrm{def}}$). All calculated values are in kcal/mol.

## Data Availability

FMO calculation was applied using FMO code version 5.1 as embedded in General Atomic and Molecular Electronic Structure System (GAMESS), available at https://www.msg.chem.iastate.edu/gamess/ (accessed on 21 May 2022). PM7/COMOS calculation was carried out through MOPAC2016, available at http://openmopac.net/ (accessed on 8 June 2022). Deformation energy calculation was performed using Molclus software, available at http://www.keinsci.com/research/molclus.html (accessed on 29 December 2020). Interaction entropy calculation was extracted from trajectories of MD simulation. The MM/PB(GB)SA calculations were performed using the Amber18 program. The molecular docking was performed using Autodock Vina 1.1.2. Calculated energy values are summarized in the Supporting Information and Supplemental Data. All complex structures and input files for FMO, FMO/SMD, FMO/PCM, and COSMO calculation in this study have been deposited in the Zenodo database under accession code 8385313.

## Supplementary Materials

The following supporting information can be downloaded at: https://www.mdpi.com/article/10.3390/ijms25010671/s1.
