# Supplementary material for: Binding Free Energy Calculation Based on the Fragment Molecular Orbital Method and Its Application in Designing Novel SHP-2 Allosteric Inhibitors
Source: Int J Mol Sci. 2024 Jan 4;25(1):671. doi: 10.3390/ijms25010671 (PMC10779950; doi:10.3390/ijms25010671)
Supplement: Supplementary file 1 [file ijms-25-00671-s001.zip › Supporting Information.pdf]

Supporting Information

Binding Free Energy Calculation Based on the Fragment Molecular Orbital Method and Its Application in Designing Novel SHP-2 Allosteric Inhibitors

Zhen Yuan [1,†], Xingyu Chen [1,†], Sisi Fan [1], Longfeng Chang [1], Linna Chu [1], Ying Zhang [1], Jie Wang [1], Shuang Li [1], Jinxin Xie [1], Jianguo Hu [1], Runyu Miao [1], Lili Zhu [1], Zhenjiang Zhao [1], Honglin Li [1,2,3,*] and Shiliang Li [1,2,*]

1 Shanghai Key Laboratory of New Drug Design, State Key Laboratory of Bioreactor Engineering, School of Pharmacy, East China University of Science & Technology, Shanghai 200237, China; y12212121@mail.ecust.edu.cn (Z.Y.); y30211246@mail.ecust.edu.cn (X.C.); y30201249@mail.ecust.edu.cn (S.F.); zhjzhao@ecust.edu.cn (Z.Z.)
2 Innovation Center for AI and Drug Discovery, East China Normal University, Shanghai 200062, China
3 Lingang Laboratory, Shanghai 200031, China
* Correspondence: hlli@ecust.edu.cn (H.L.); slli@hsc.ecnu.edu.cn (S.L.)
† These authors contributed equally to this work.

Table S1. Results for ranking with different affinity prediction methods in Schrödinger FEP+ benchmark set.

	FMO					FMOScore					FEP+				
Protein	N	R	ρ	τ	RMSE	N	R	ρ	τ	RMSE	N	R	ρ	τ	RMSE
CDK8	32	$0.78^{0.71}_{0.85}$	$0.82^{0.73}_{0.89}$	$0.64^{0.52}_{0.73}$	$0.93^{0.64}_{1.26}$	32	$0.81^{0.72}_{0.88}$	$0.84^{0.75}_{0.92}$	$0.67^{0.56}_{0.78}$	$0.88^{0.63}_{1.15}$	32	$0.64^{0.44}_{0.81}$	$0.67^{0.46}_{0.84}$	$0.53^{0.36}_{0.69}$	$1.11^{0.66}_{1.55}$
c-Met	24	$0.83^{0.72}_{0.91}$	$0.79^{0.60}_{0.92}$	$0.65^{0.47}_{0.80}$	$1.04^{0.71}_{1.37}$	24	$0.90^{0.82}_{0.96}$	$0.90^{0.79}_{0.97}$	$0.77^{0.64}_{0.88}$	$0.78^{0.43}_{1.09}$	24	$0.91^{0.85}_{0.95}$	$0.88^{0.77}_{0.95}$	$0.75^{0.63}_{0.85}$	$0.76^{0.57}_{0.97}$
Eg5	28	$0.68^{0.48}_{0.83}$	$0.59^{0.31}_{0.81}$	$0.56^{0.22}_{0.65}$	$0.69^{0.47}_{0.91}$	28	$0.79^{0.66}_{0.88}$	$0.74^{0.54}_{0.89}$	$0.60^{0.41}_{0.76}$	$0.58^{0.41}_{0.76}$	28	$0.73^{0.57}_{0.86}$	$0.71^{0.52}_{0.86}$	$0.57^{0.40}_{0.73}$	$0.68^{0.47}_{0.92}$
HIF-2α	42	$0.31^{0.05}_{0.54}$	$0.23^{-0.05}_{0.49}$	$0.16^{-0.03}_{0.36}$	$1.12^{0.78}_{1.46}$	42	$0.43^{0.13}_{0.67}$	$0.28^{0.01}_{0.53}$	$0.21^{0.01}_{0.40}$	$1.07^{0.77}_{1.38}$	42	$0.57^{0.23}_{0.80}$	$0.54^{0.29}_{0.74}$	$0.41^{0.22}_{0.58}$	$0.98^{0.62}_{1.34}$
PFKFB3	40	$0.86^{0.80}_{0.91}$	$0.84^{0.75}_{0.90}$	$0.77^{0.56}_{0.76}$	$0.56^{0.42}_{0.69}$	40	$0.90^{0.85}_{0.93}$	$0.89^{0.81}_{0.94}$	$0.73^{0.63}_{0.81}$	$0.49^{0.37}_{0.63}$	40	$0.81^{0.73}_{0.88}$	$0.81^{0.71}_{0.89}$	$0.64^{0.53}_{0.74}$	$0.64^{0.47}_{0.80}$
SHP-2	26	$0.57^{0.34}_{0.74}$	$0.59^{0.33}_{0.79}$	$0.45^{0.26}_{0.63}$	$1.05^{0.76}_{1.39}$	26	$0.80^{0.70}_{0.88}$	$0.75^{0.56}_{0.88}$	$0.56^{0.39}_{0.70}$	$0.76^{0.50}_{1.02}$	26	$0.73^{0.58}_{0.85}$	$0.71^{0.50}_{0.87}$	$0.57^{0.39}_{0.73}$	$0.88^{0.64}_{1.15}$
SYK	39	$0.43^{0.27}_{0.57}$	$0.43^{0.21}_{0.63}$	$0.31^{0.15}_{0.27}$	$0.77^{0.57}_{0.96}$	39	$0.76^{0.62}_{0.87}$	$0.76^{0.61}_{0.88}$	$0.61^{0.46}_{0.74}$	$0.54^{0.37}_{0.72}$	39	$0.47^{0.18}_{0.70}$	$0.40^{0.16}_{0.60}$	$0.28^{0.11}_{0.43}$	$0.75^{0.56}_{0.94}$
TNKS2	27	$0.36^{0.05}_{0.58}$	$0.21^{-0.19}_{0.54}$	$0.10^{-0.19}_{0.37}$	$1.02^{0.65}_{1.35}$	27	$0.62^{0.29}_{0.85}$	$0.50^{0.17}_{0.77}$	$0.39^{0.12}_{0.63}$	$0.86^{0.51}_{1.19}$	27	$0.46^{0.16}_{0.70}$	$0.43^{0.10}_{0.69}$	$0.32^{0.07}_{0.54}$	$0.98^{0.66}_{1.29}$
Total	258	$0.62^{0.55}_{0.68}$	$0.64^{0.57}_{0.70}$	$0.47^{0.41}_{0.52}$	$1.14^{1.02}_{1.26}$	258	$0.87^{0.84}_{0.89}$	$0.87^{0.84}_{0.89}$	$0.69^{0.65}_{0.72}$	$0.71^{0.62}_{0.80}$	258	$0.78^{0.74}_{0.83}$	$0.77^{0.71}_{0.81}$	$0.58^{0.53}_{0.63}$	$0.89^{0.77}_{1.00}$

	MM/GBSA					MM/PBSA					Vina				
Protein	N	R	ρ	τ	RMSE	N	R	ρ	τ	RMSE	N	R	ρ	τ	RMSE
CDK8	32	$0.30^{0.03}_{0.68}$	$0.45^{0.16}_{0.71}$	$0.33^{0.11}_{0.53}$	$1.42^{0.93}_{2.09}$	32	$0.02^{-0.34}_{0.38}$	$-0.04^{-0.34}_{0.38}$	$-0.02^{-0.27}_{0.24}$	$1.50^{1.02}_{2.03}$	32	$0.58^{0.35}_{0.79}$	$0.61^{0.36}_{0.80}$	$0.48^{0.27}_{0.66}$	$1.17^{0.73}_{1.70}$
c-Met	24	$0.68^{0.48}_{0.83}$	$0.72^{0.47}_{0.89}$	$0.59^{0.39}_{0.77}$	$1.43^{0.89}_{2.02}$	24	$0.23^{-0.16}_{0.56}$	$0.15^{-0.25}_{0.51}$	$0.10^{-0.19}_{0.39}$	$1.83^{1.26}_{2.46}$	24	$-0.31^{-0.56}_{-0.05}$	$-0.37^{-0.62}_{-0.07}$	$-0.22^{-0.44}_{0}$	$1.80^{1.26}_{2.32}$
Eg5	28	$0.43^{0.11}_{0.69}$	$0.41^{0.08}_{0.70}$	$0.30^{0.04}_{0.53}$	$0.88^{0.61}_{1.17}$	28	$0.02^{-0.27}_{0.31}$	$0.10^{-0.25}_{0.43}$	$0.05^{-0.20}_{0.29}$	$0.96^{0.65}_{1.27}$	28	$-0.44^{-0.65}_{-0.22}$	$-0.47^{-0.69}_{-0.20}$	$-0.31^{-0.50}_{-0.10}$	$0.84^{0.51}_{1.15}$
HIF-2α	42	$0.19^{-0.03}_{0.43}$	$0.23^{0.0003}_{0.45}$	$0.15^{-0.005}_{0.30}$	$1.15^{0.82}_{1.52}$	42	$0.26^{0.001}_{0.51}$	$0.20^{-0.06}_{0.45}$	$0.15^{-0.04}_{0.33}$	$1.14^{0.80}_{1.49}$	42	$0.28^{0.003}_{0.54}$	$0.50^{0.26}_{0.70}$	$0.39^{0.21}_{0.56}$	$1.13^{0.76}_{1.50}$
PFKFB3	40	$0.48^{0.23}_{0.70}$	$0.45^{0.19}_{0.69}$	$0.33^{0.14}_{0.51}$	$0.99^{0.73}_{1.28}$	40	$0.26^{0.01}_{0.49}$	$0.28^{0.005}_{0.54}$	$0.21^{0.01}_{0.41}$	$1.10^{0.87}_{1.34}$	40	$0.35^{0.13}_{0.54}$	$0.33^{0.10}_{0.54}$	$0.22^{0.05}_{0.38}$	$1.06^{0.84}_{1.28}$
SHP-2	26	$0.34^{0.08}_{0.64}$	$0.35^{0.02}_{0.67}$	$0.31^{0.08}_{0.55}$	$1.24^{0.82}_{1.75}$	26	$0.50^{0.19}_{0.77}$	$0.48^{0.18}_{0.74}$	$0.38^{0.15}_{0.59}$	$1.19^{0.70}_{1.73}$	26	$0.37^{0.04}_{0.67}$	$0.35^{0.04}_{0.64}$	$0.35^{0.01}_{0.48}$	$1.17^{0.72}_{1.61}$
SYK	39	$0.50^{0.29}_{0.69}$	$0.47^{0.24}_{0.68}$	$0.36^{0.19}_{0.52}$	$0.74^{0.55}_{0.93}$	39	$0.18^{-0.12}_{0.43}$	$0.06^{-0.23}_{0.34}$	$0.04^{-0.16}_{0.24}$	$0.84^{0.61}_{1.06}$	39	$0.39^{0.21}_{0.56}$	$0.35^{0.14}_{0.54}$	$0.34^{0.09}_{0.37}$	$0.77^{0.55}_{0.99}$
TNKS2	27	$0.47^{0.19}_{0.69}$	$0.41^{0.13}_{0.65}$	$0.28^{0.07}_{0.48}$	$0.96^{0.63}_{1.27}$	27	$-0.21^{-0.48}_{0.07}$	$-0.11^{-0.43}_{0.20}$	$-0.09^{-0.31}_{0.12}$	$1.06^{0.68}_{0.36}$	27	$-0.06^{-0.35}_{0.25}$	$0.11^{-0.22}_{0.44}$	$0.09^{-0.14}_{0.33}$	$1.10^{0.70}_{1.44}$
Total	258	$0.27^{0.18}_{0.36}$	$0.27^{0.17}_{0.26}$	$0.18^{0.12}_{0.25}$	$1.39^{1.25}_{1.54}$	258	$0.13^{0.03}_{0.23}$	$0.09^{-0.01}_{0.19}$	$0.06^{-0.01}_{0.19}$	$1.44^{1.30}_{1.57}$	258	$0.09^{0.008}_{0.18}$	$0.09^{0.00}_{0.18}$	$0.06^{0.00}_{0.12}$	$1.44^{1.30}_{1.57}$

Table S2. Linear coefficients of FMOScore of 8 targets in Schrödinger FEP+ benchmark.

Target	ΔE^{int}	ΔG^{sol}	ΔE^{def}_{lig}	constant
CDK8	0.05066	-0.04087	0.02395	-3.05111
c-Met	0.04297	0.03001	0.04486	-3.85653
EG5	0.04591	-0.00677	0.01301	-6.73679
HIF-2α	0.02229	0.01377	0.07526	-8.75932
PFKFB3	0.05389	-0.00547	0.03022	-3.05507
SHP2	0.02002	-0.02125	0.04421	-2.78159
SYK	0.01577	-0.0056	0.04735	-9.47309
TNKS2	0.08894	-0.03104	0.05194	-1.31775

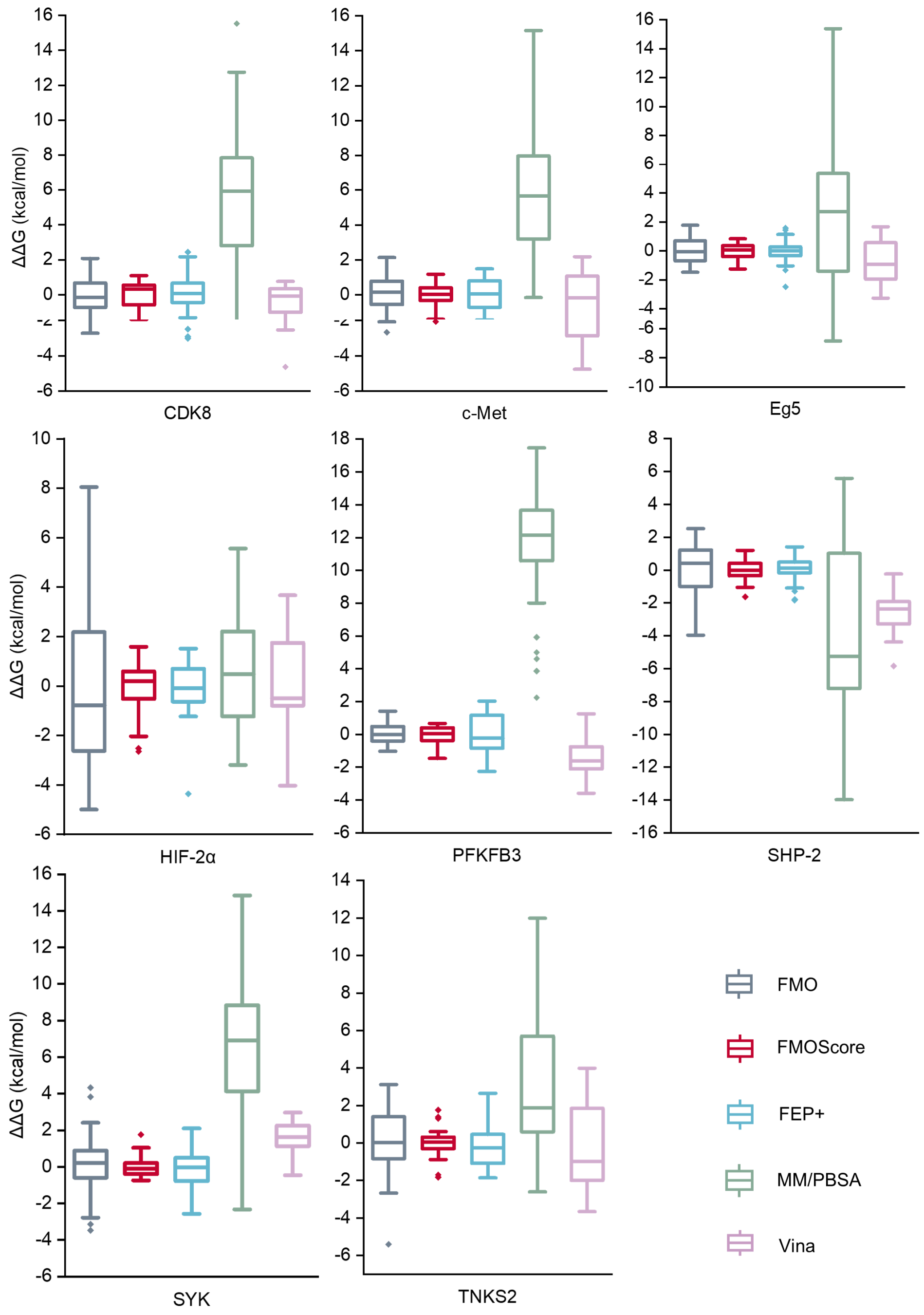

Figure S1. Box-plot diagrams of the error for pairwise relative affinities |ΔG_{pred} – ΔG_{exp}|.

Table S3. Results of different energy term contributions for eight targets with the FMOScore method on a literature curated benchmark set.

Protein	PDB ID [a]	ΔG_{exp} [b]	ΔG_{pred} [c]	ΔE^{int}					ΔG^{sol}	ΔE^{def}_{lig}
				Total [d]	ΔE_{es}	ΔE_{ex}	ΔE_{ct+mix}	ΔE_{disp}		
CDK8	5HNB	-11.22	-11.86	-129.11	-93.83	61.14	-26.82	-69.60	61.73	12.63
c-Met	4R1Y	-8.73	-10.79	-174.79	-121.22	62.26	-28.53	-87.30	-30.36	32.47
Eg5	3L9H	-11.05	-11.04	-91.23	-43.25	58.82	-25.41	-81.39	119.70	44.91
HIF-2α	5TBM	-11.01	-10.54	-135.58	-92.19	76.45	-32.98	-86.86	43.36	7.74
PFKFB3	6HV1	-10.3	-10.15	-134.46	-87.75	72.19	-28.62	-90.27	53.92	10.37
SHP-2	5EHR	-9.75	-9.76	-141.73	-131.02	98.16	-30.97	-77.90	196.22	7.55
SYK	4PV0	-11.42	-10.75	-132.67	-94.44	62.87	-24.57	-76.53	69.14	26.54
TNKS2	4UI5	-10.05	-10.93	-106.69	-68.45	69.01	-27.56	-79.69	46.88	14.78

[a]The structures of the lead compounds in each target in the FEP+ benchmark have been selected for presentation, which are usually the starting points for structural modifications. [b]Experimental affinities were converted to ΔG_{exp} values using the equation $\Delta G_{exp} \approx k_B T \log IC_{50}$. [c]Predicted binding free energy ΔG_{prep} was fitted to the three types of energies calculated by the FMOScore method, consisting of binding affinities in vacuo (ΔE^{int}), solvation free energy (ΔG^{sol}), and deformation energy into the isolated form (ΔE^{def}_{lig}). [d]The total fragment pair interaction energy (ΔE^{int}) was decomposed into the electrostatic term (ΔE_{es}), exchange-repulsion term (ΔE_{ex}), charge transfer terms (ΔE_{ct+mix}), and dispersion term (ΔE_{disp}). All calculated values are in kcal/mol.

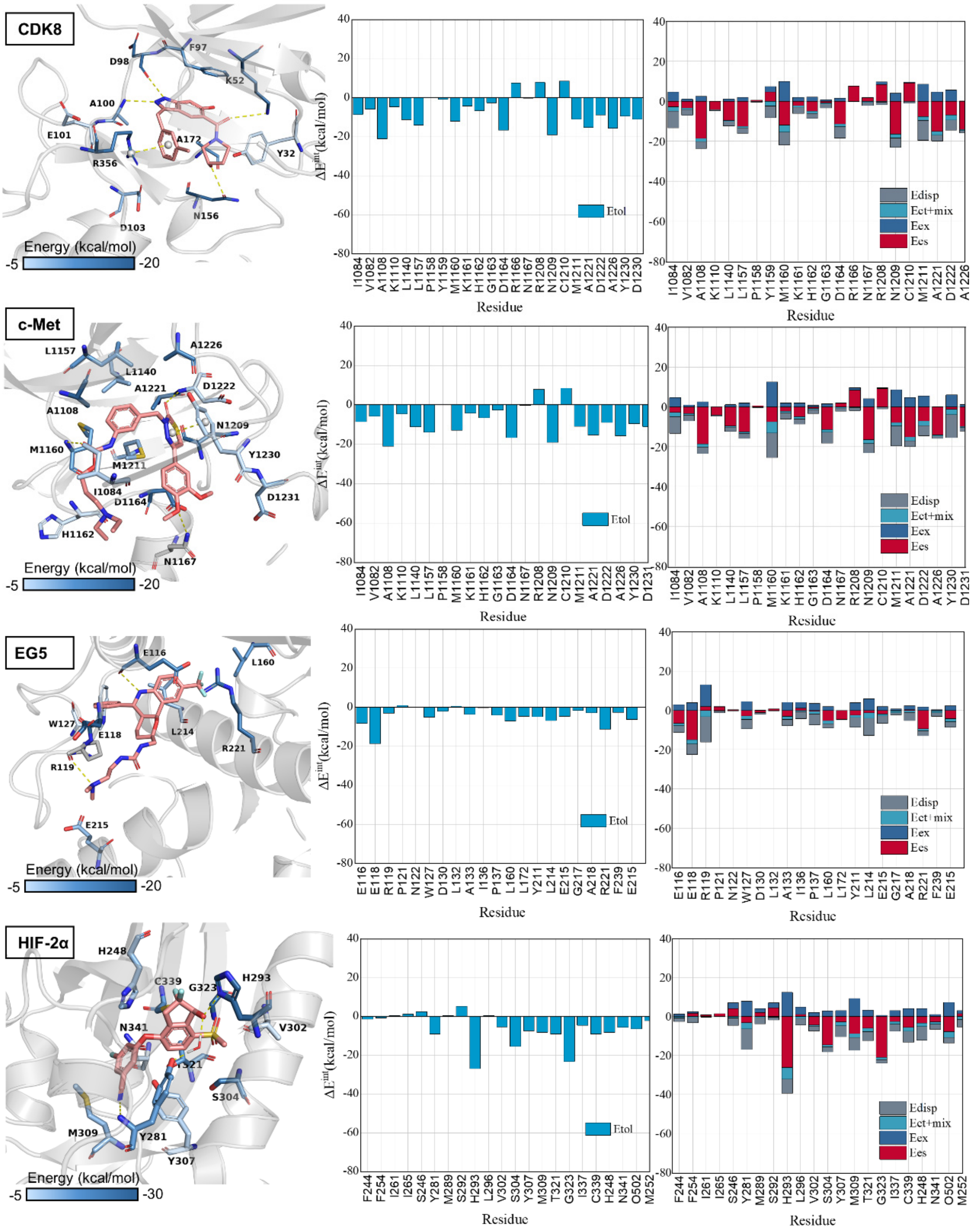
CDK8
D98
F97
K52
A100
E101
A172
Y32
R356
D103
N156
Energy (kcal/mol)
-5
-20
ΔE^int(kcal/mol)
Etol
Edisp
Ect+mix
Eex
Ees
Residue
I1084 V1082 A1108 K1110 L1140 L1157 P1158 Y1159 M1160 K1161 H1162 G1163 D1164 R1166 N1167 R1208 N1209 C1210 M1211 A1221 D1222 A1226 Y1230 D1230
c-Met
L1157
A1226
L1140
A1221
D1222
A1108
N1209
M1160
M1211
Y1230
I1084
D1164
D1231
H1162
N1167
Energy (kcal/mol)
-5
-20
I1084 V1082 A1108 K1110 L1140 L1157 P1158 M1160 K1161 H1162 G1163 D1164 N1167 R1208 N1209 C1210 M1211 A1221 D1222 A1226 Y1230 D1231
EG5
E116
L160
W127
E118
L214
R221
R119
E215
Energy (kcal/mol)
-5
-20
E116 E118 R119 P121 N122 W127 D130 L132 A133 I136 P137 L160 L172 Y211 L214 E215 G217 A218 R221 F239 E215
HIF-2α
H248
C339
G323
H293
N341
V302
T321
S304
M309
Y281
Y307
Energy (kcal/mol)
-5
-30
F244 F254 I261 I265 S246 Y281 M289 S292 H293 L296 V302 S304 Y307 M309 T321 G323 I337 C339 H248 N341 O502 M252

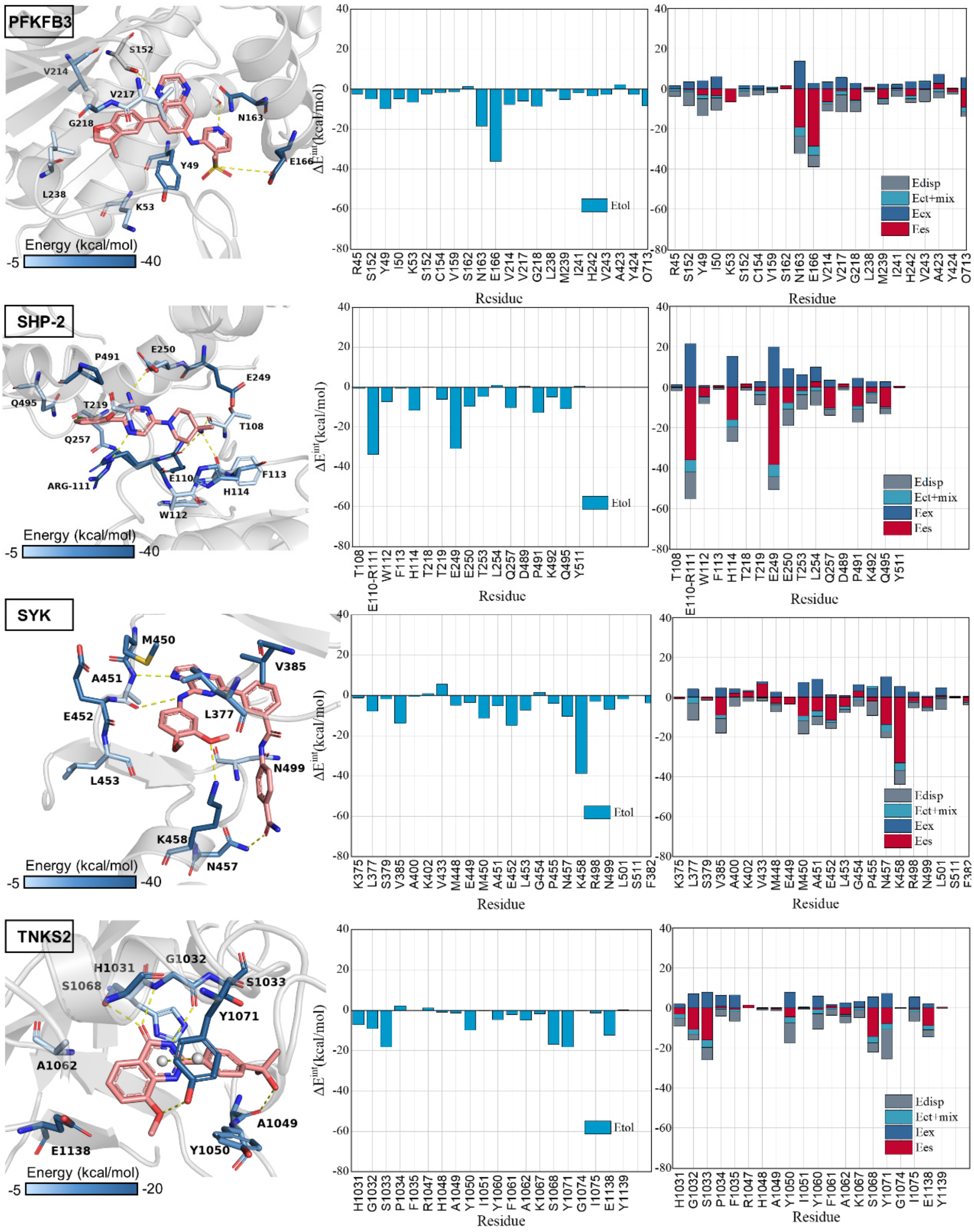

Figure S2. FMO results of eight targets: CDK8 (PDB: 5HNB), c-Met (PDB: 4R1Y), EG5 (PDB: 3I9H), HIF-2α (PDB: 5TBM), PFKFB3 (PDB: 6HVJ), SHP-2 (PDB: 5EHR), SYK (PDB: 4PV0), TNKS2 (PDB: 4UI5). The key interactions according to FMO calculations are marked as yellow dashed lines, and key residues are shown in dark blue sticks. The left-hand bar plots describe PIE between the residues at the active site and ligands, and the right-hand plots show the PIEDA of these key interactions.

The electrostatics, charge-transfer, dispersion, and exchange-repulsion PIE terms are represented in red, blue, gray, and dark blue, respectively.

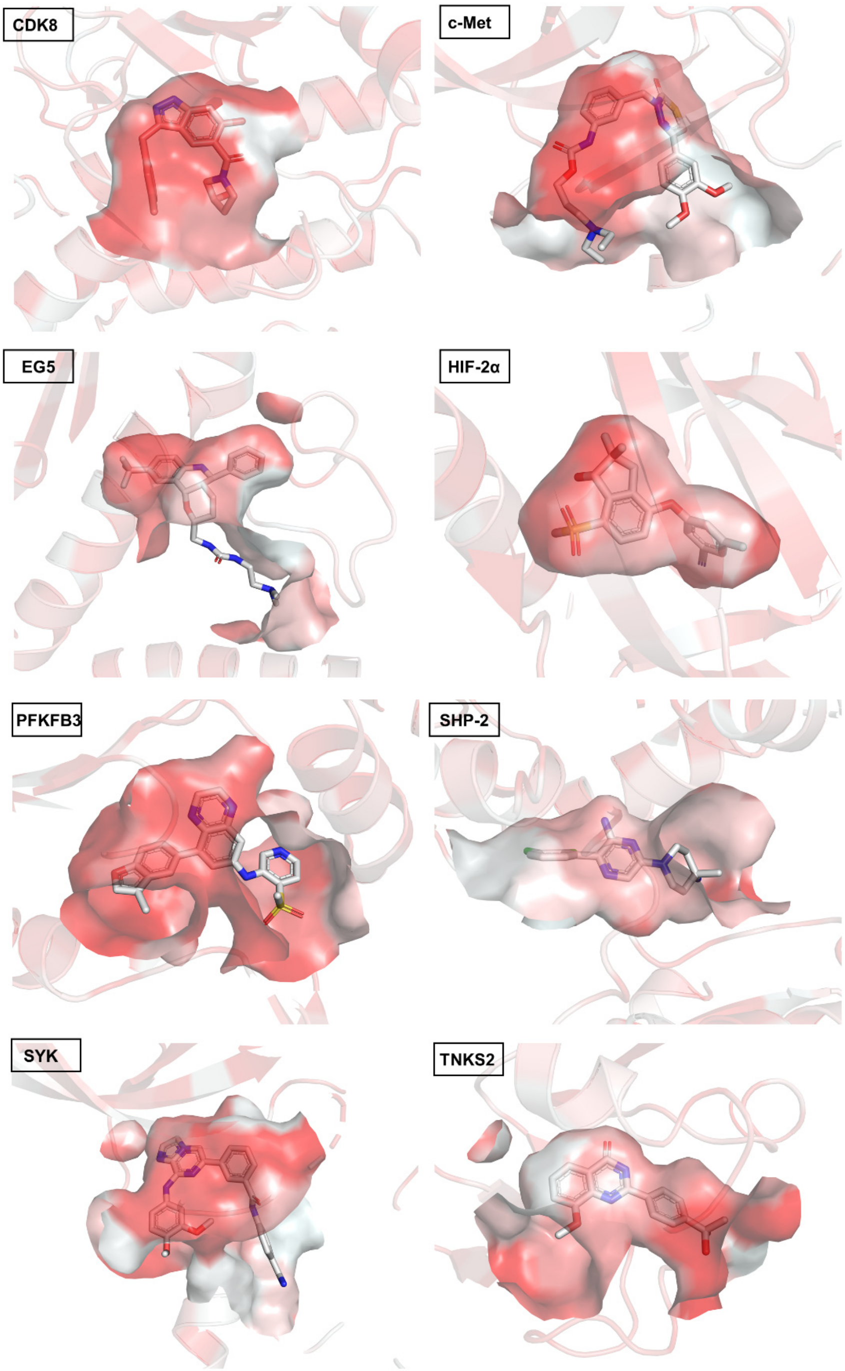

Figure S3. Pocket shapes and hydrophobic surfaces were rendered with PyMOL (version 2.3.0) for the eight targets: CDK8 (PDB: 5HNB), c-Met (PDB: 4R1Y), EG5 (PDB: 3I9H), HIF-2α (PDB: 5TBM), PFKFB3 (PDB: 6HVJ), SHP-2 (PDB: 5EHR), SYK (PDB: 4PV0), TNKS2 (PDB: 4UI5). The redder color indicates a stronger hydrophobic surface.

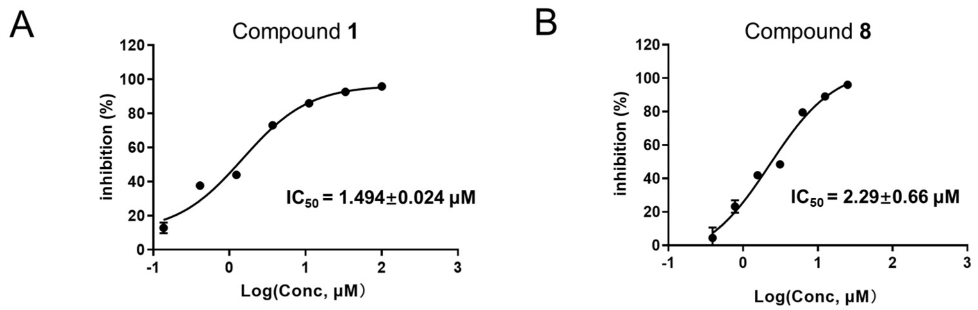

Figure S4. Identification of SHP-2 inhibitors through biochemical screening. (A-B) Compound **1** and **8** exhibited potent inhibitory activity against SHP-2. Phosphatase assays were conducted using the artificial substrate DIFMUP, and data represent mean ± SD (n = 3).

Table S4. Structure activity relationship study of THR-β inhibition activities.

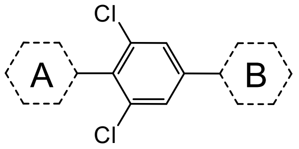

Ligand	Part A	Part B	THR-β IC_{50} (μM) [a]	ΔG_{exp} [b]	FMOScore (kcal/mol)			
					ΔG_{pred} [c]	ΔE^{int}	ΔE^{def}_{lig}	ΔG^{sol}
**1**			0.99±0.08	-8.186	-9.629	-101.46	27.19	245.55
**2**			>33	-6.110	-6.318	-80.016	24.10	224.18
**3**			0.65±0.07	-8.435	-8.475	-105.35	18.56	236.24
**4**			>100	-5.453	-5.925	-84.49	16.06	224.85

**5**	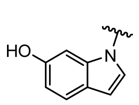	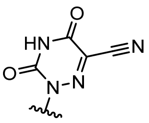	>100	-5.453	-6.624	-78.58	19.95	236.48
**6**	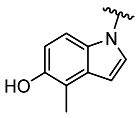	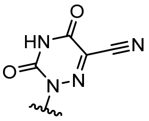	0.03±0.01	-10.257	-9.177	-112.88	22.27	232.94
**7**	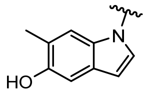	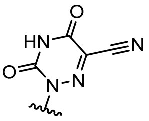	>3.7	-7.406	-7.832	-95.74	22.45	231.71
**8**	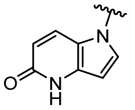	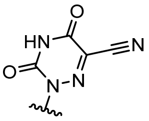	>1.2	-8.072	-7.740	-91.58	20.76	237.49
**9**	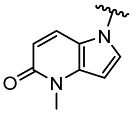	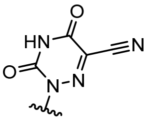	0.20±0.05	-9.133	-9.539	-107.01	21.70	245.91
**10**	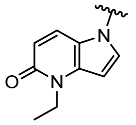	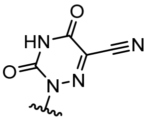	0.02±0.001	-10.497	-9.468	-101.48	25.28	245.88
**11**	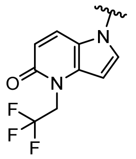	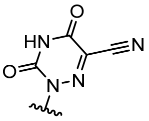	0.07±0.01	-9.755	-9.921	-106.95	26.54	244.73
**12**	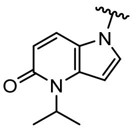	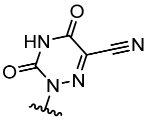	0.03±0.01	-10.257	-10.135	-107.75	27.03	246.33
**13**	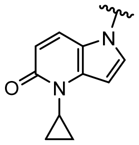	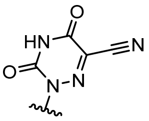	0.03±0.007	-10.257	-9.983	-110.78	27.52	239.90
**14**	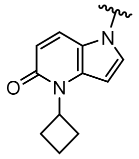	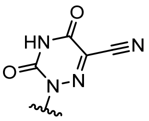	0.02±0.006	-10.497	-10.126	-100.10	32.65	246.69
**15**	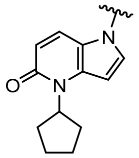	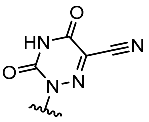	0.04±0.01	-10.086	-10.282	-106.87	35.74	236.90

**16**	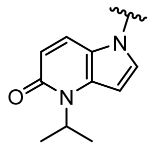	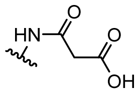	0.21±0.06	-9.105	-10.820	-99.17	30.21	261.81
**17**	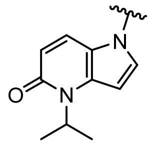	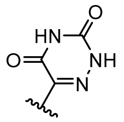	0.04±0.007	-10.086	-7.824	-75.72	24.64	251.07
**18**	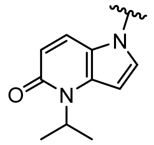	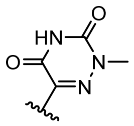	0.009±0.003	-10.970	-10.192	-108.04	28.32	245.01
	R^2		-	-	0.71	0.46	0.40	0.37

The spectra of ^{1}H NMR, ^{13}C NMR and HRMS (ESI) of all final compounds

Compound 1

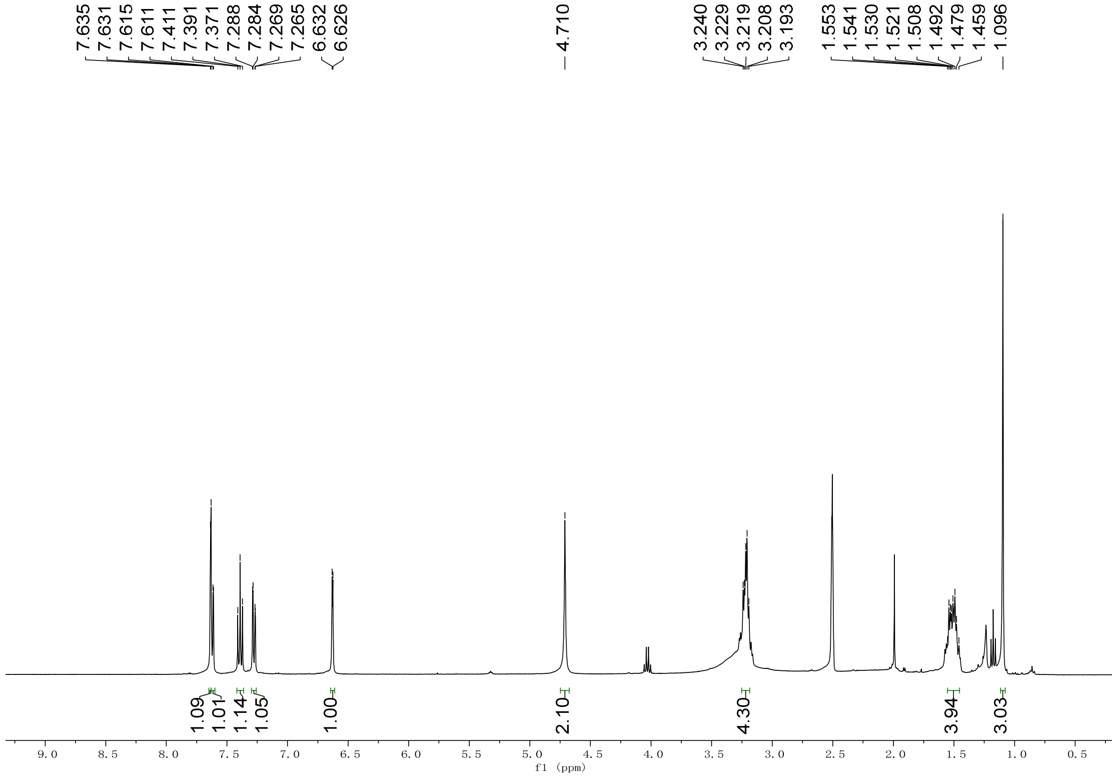

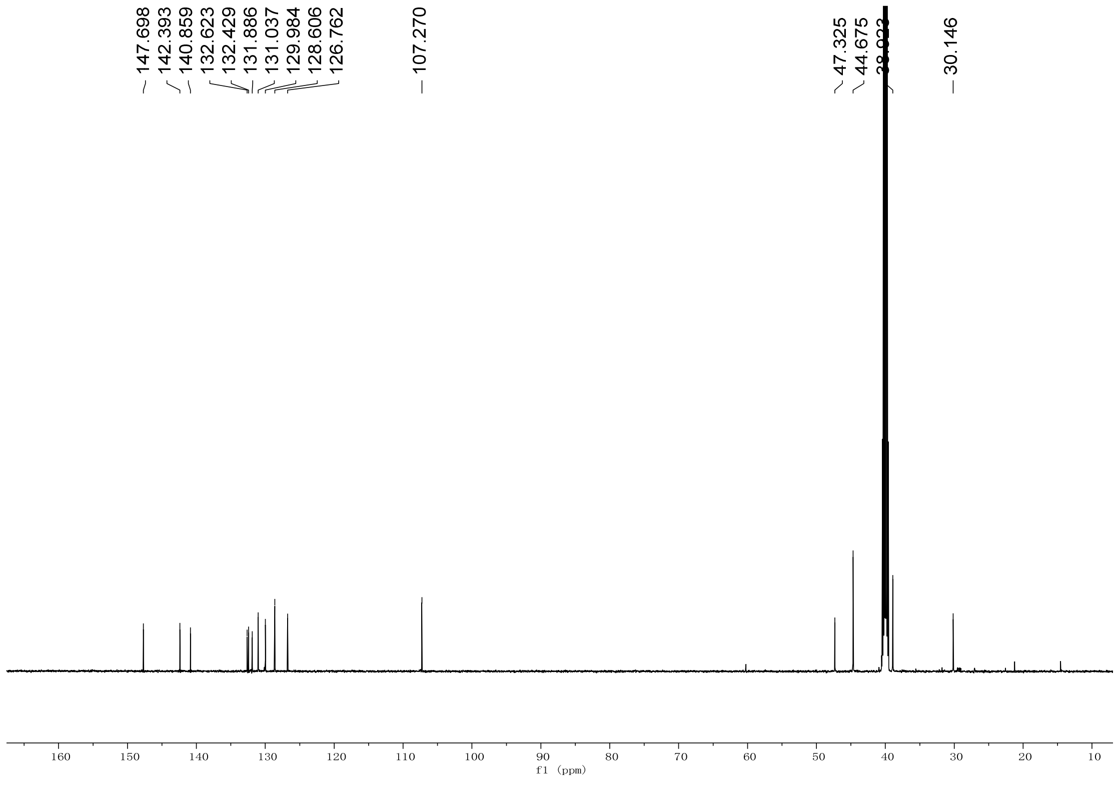

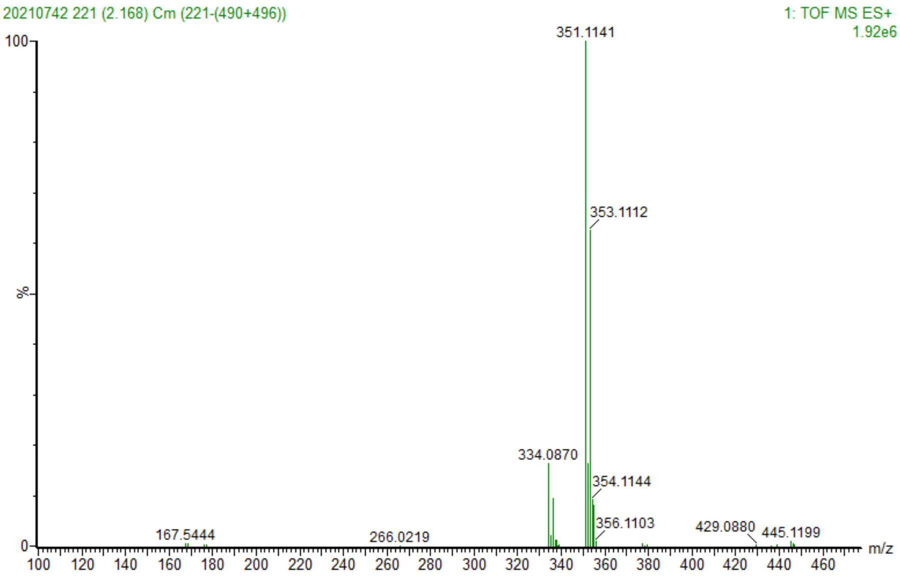
20210742 221 (2.168) Cm (221-(490+496))
1: TOF MS ES+
1.92e6
351.1141
353.1112
334.0870
354.1144
356.1103
167.5444
266.0219
429.0880
445.1199
%
m/z

Compound 2

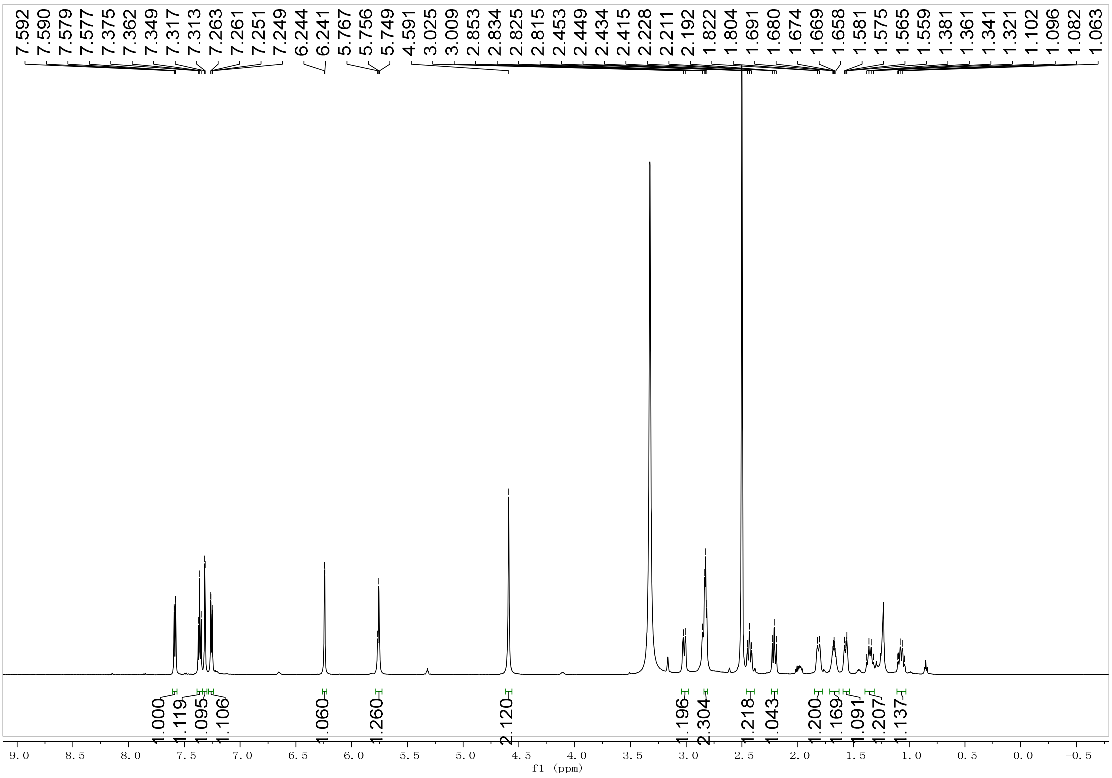
7.592
7.590
7.579
7.577
7.375
7.362
7.349
7.317
7.313
7.263
7.261
7.251
7.249
6.244
6.241
5.767
5.756
5.749
4.591
3.025
3.009
2.853
2.834
2.825
2.815
2.453
2.449
2.434
2.415
2.228
2.211
2.192
1.822
1.804
1.691
1.680
1.674
1.669
1.658
1.581
1.575
1.565
1.559
1.381
1.361
1.341
1.321
1.102
1.096
1.082
1.063
1.000
1.119
1.095
1.106
1.060
1.260
2.120
1.196
2.304
1.218
1.043
1.200
1.169
1.091
1.207
1.137
f1 (ppm)

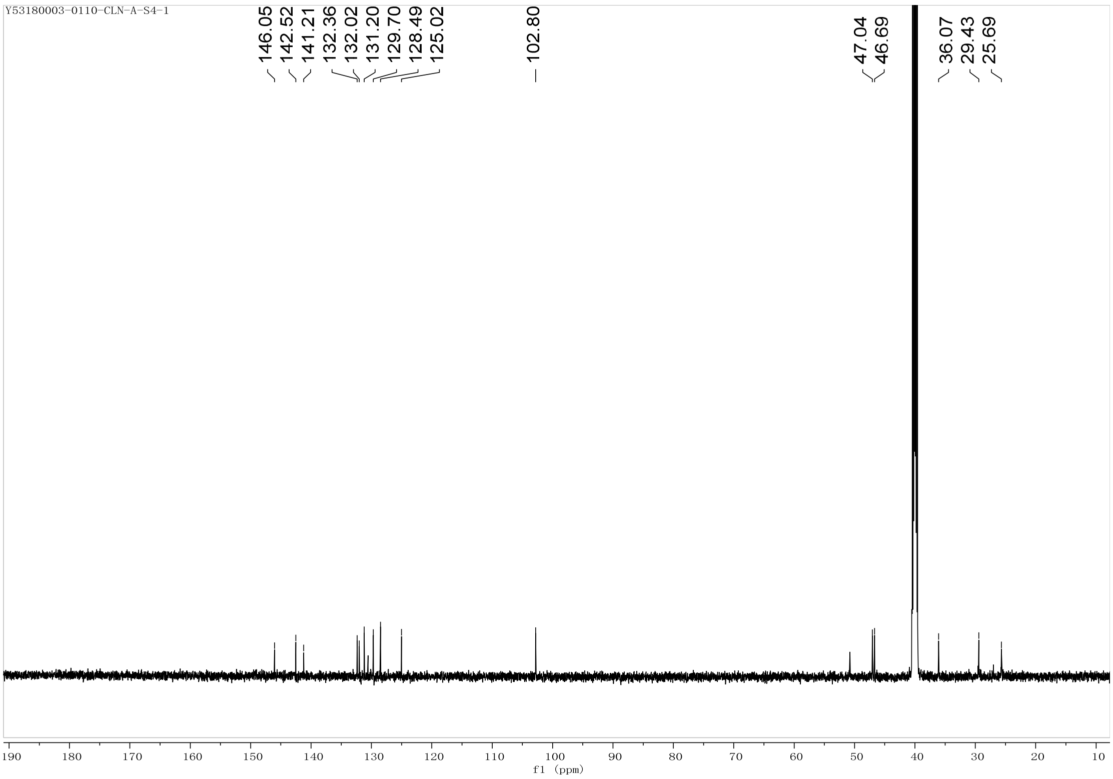

Y53180003-0110-CLN-A-S4-1
146.05
142.52
141.21
132.36
132.02
131.20
129.70
128.49
125.02
102.80
47.04
46.69
36.07
29.43
25.69
190 180 170 160 150 140 130 120 110 100 90 80 70 60 50 40 30 20 10
f1 (ppm)

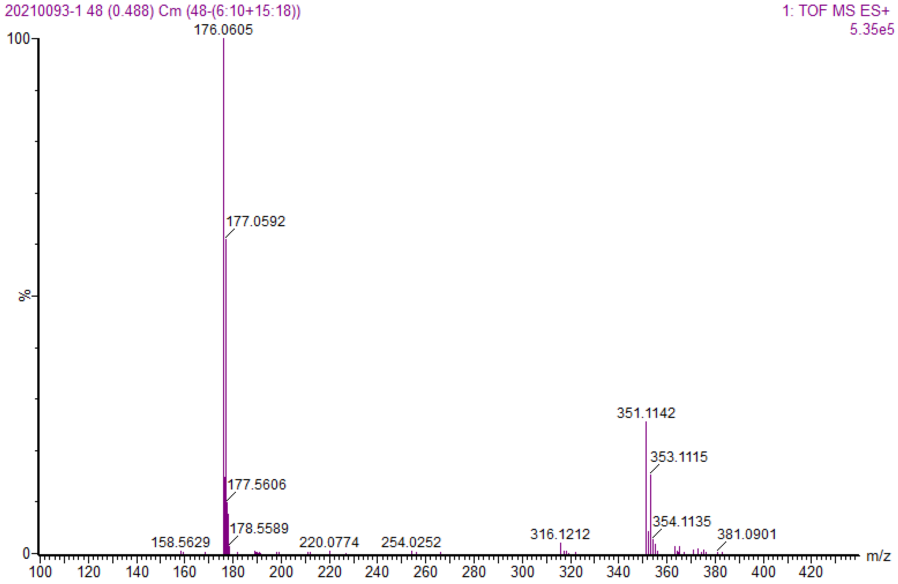

20210093-1 48 (0.488) Cm (48-(6:10+15:18))
1: TOF MS ES+
5.35e5
176.0605
177.0592
177.5606
178.5589
158.5629
220.0774
254.0252
316.1212
351.1142
353.1115
354.1135
381.0901
100
%
0
m/z
100 120 140 160 180 200 220 240 260 280 300 320 340 360 380 400 420

Compound 3

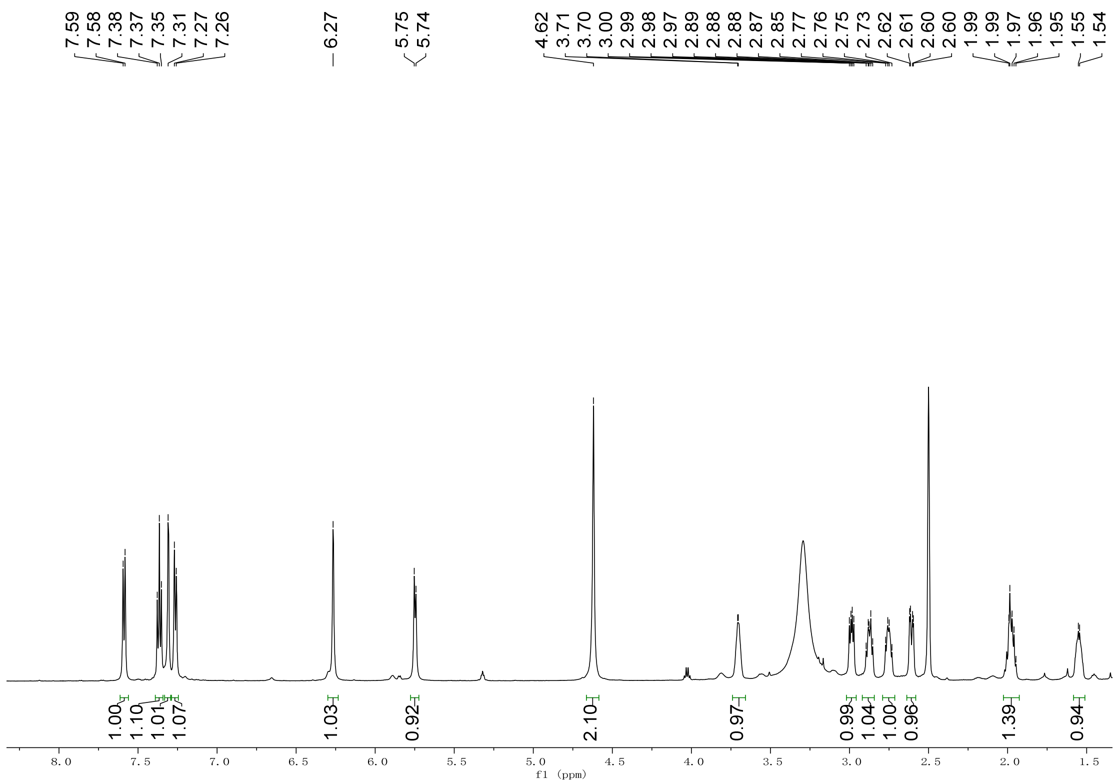
7.59
7.58
7.38
7.37
7.35
7.31
7.27
7.26
6.27
5.75
5.74
4.62
3.71
3.70
3.00
2.99
2.98
2.97
2.89
2.88
2.88
2.87
2.85
2.77
2.76
2.75
2.73
2.62
2.61
2.60
2.60
1.99
1.97
1.96
1.95
1.55
1.54
1.00
1.10
1.01
1.07
1.03
0.92
2.10
0.97
0.99
1.04
1.00
0.96
1.39
0.94
8.0 7.5 7.0 6.5 6.0 5.5 5.0 4.5 4.0 3.5 3.0 2.5 2.0 1.5
f1 (ppm)

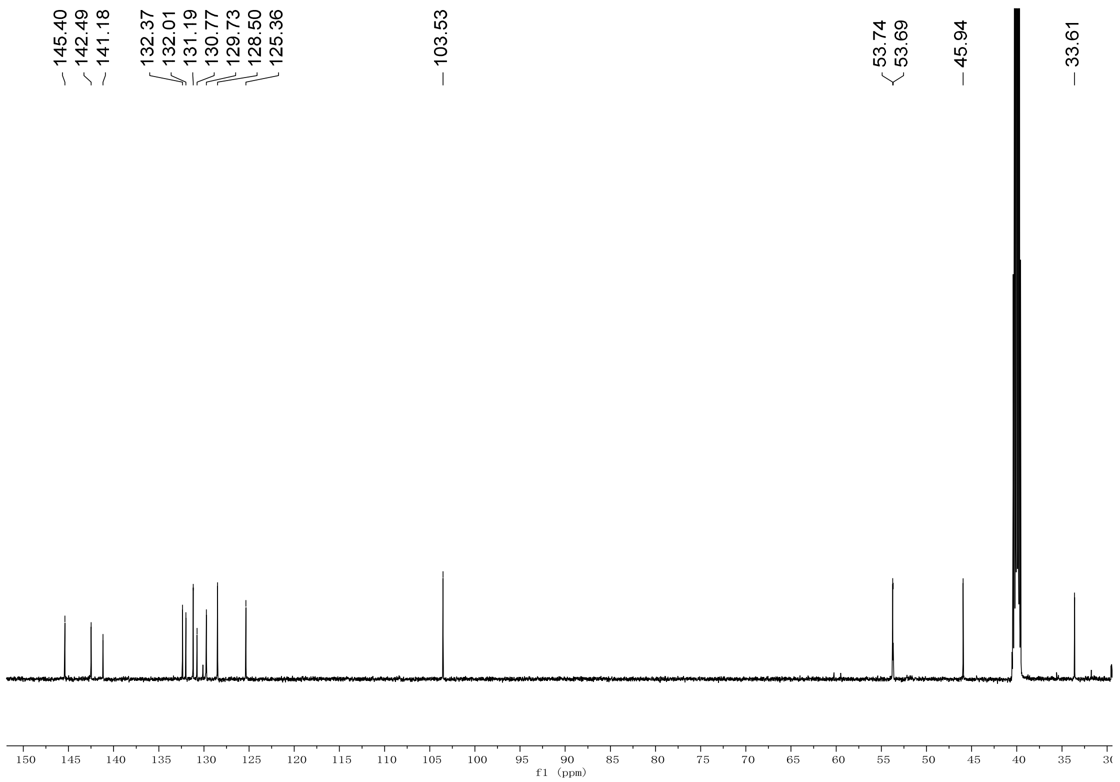
145.40
142.49
141.18
132.37
132.01
131.19
130.77
129.73
128.50
125.36
103.53
53.74
53.69
45.94
33.61
150 145 140 135 130 125 120 115 110 105 100 95 90 85 80 75 70 65 60 55 50 45 40 35 30
f1 (ppm)

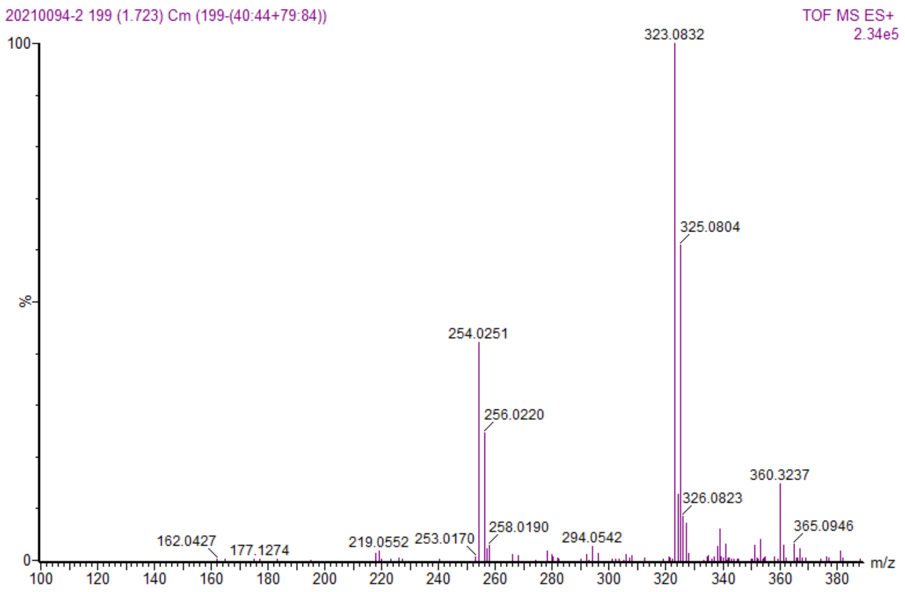
20210094-2 199 (1.723) Cm (199-(40:44+79:84))
TOF MS ES+
2.34e5
323.0832
325.0804
254.0251
256.0220
360.3237
326.0823
365.0946
258.0190
294.0542
162.0427
177.1274
219.0552
253.0170
%
m/z

Compound：4

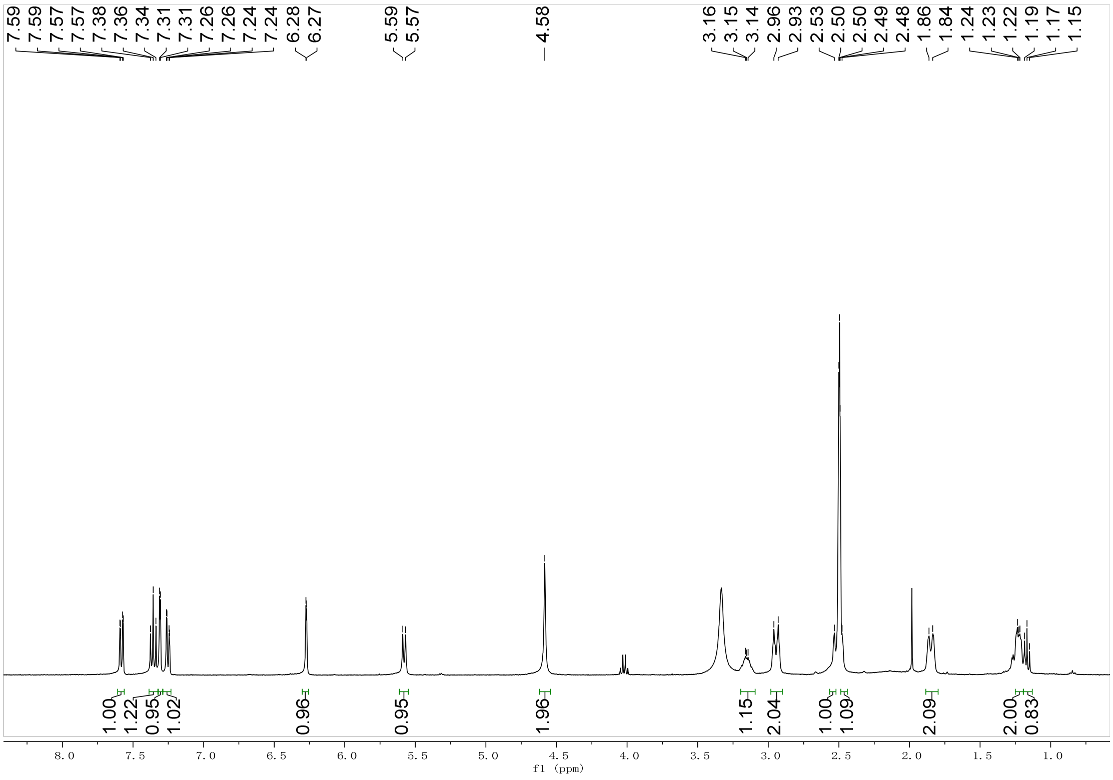
7.59 7.59 7.57 7.57 7.38 7.36 7.34 7.31 7.31 7.26 7.26 7.24 7.24 6.28 6.27 5.59 5.57 4.58 3.16 3.15 3.14 2.96 2.93 2.53 2.50 2.50 2.49 2.48 1.86 1.84 1.24 1.23 1.22 1.19 1.17 1.15
1.00 1.22 0.95 1.02 0.96 0.95 1.96 1.15 2.04 1.00 1.09 2.09 2.00 0.83
f1 (ppm)

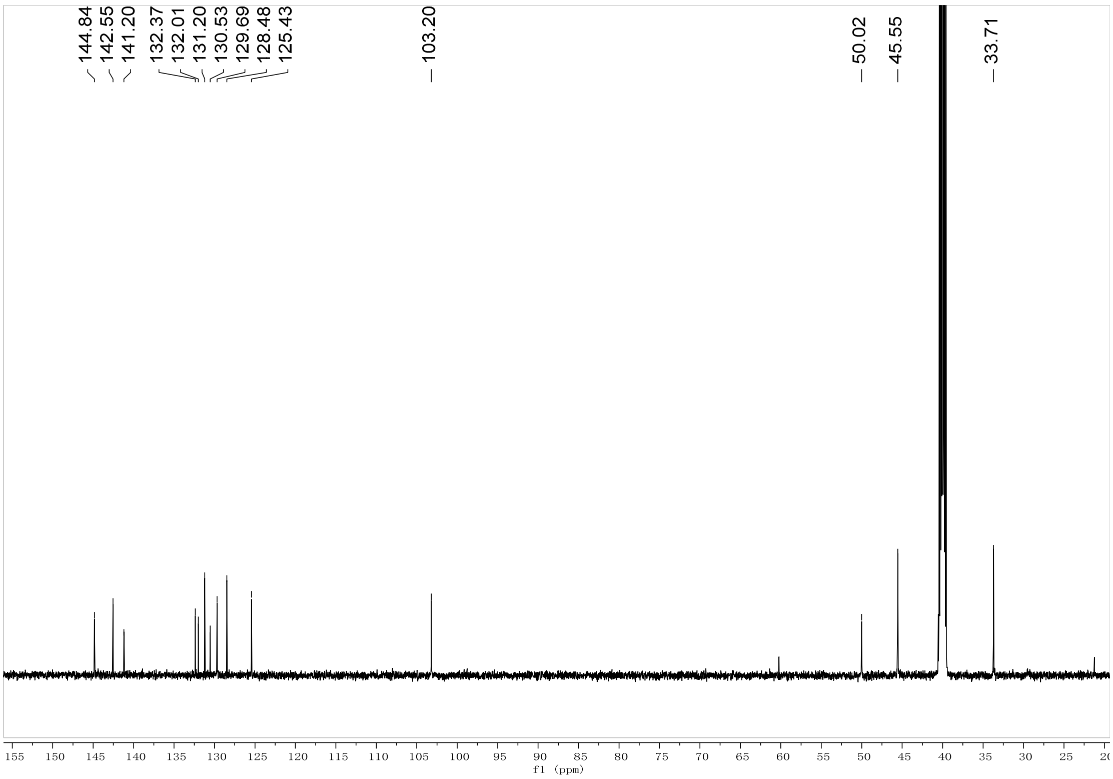
144.84
142.55
141.20
132.37
132.01
131.20
130.53
129.69
128.48
125.43
103.20
50.02
45.55
33.71
155 150 145 140 135 130 125 120 115 110 105 100 95 90 85 80 75 70 65 60 55 50 45 40 35 30 25
f1 (ppm)

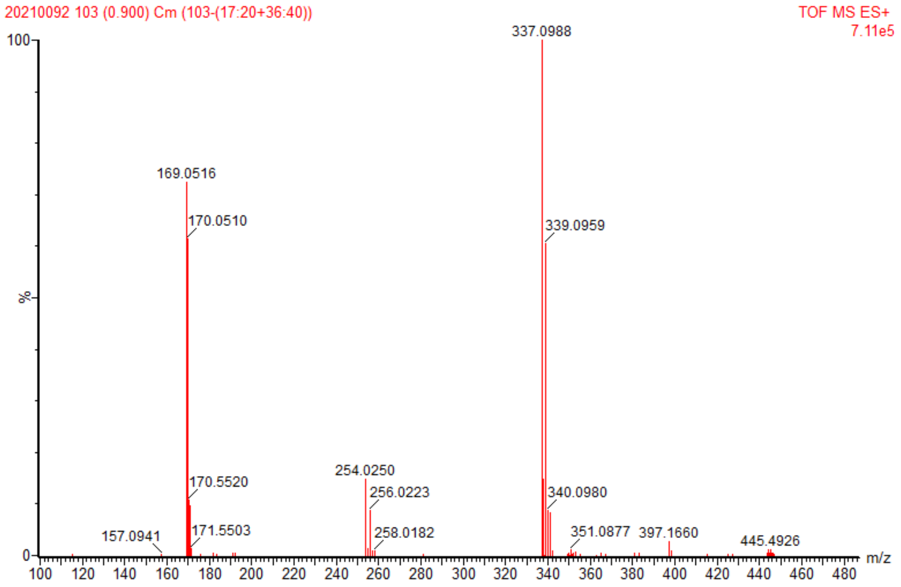
20210092 103 (0.900) Cm (103-(17:20+36:40))
TOF MS ES+
7.11e5
337.0988
169.0516
170.0510
339.0959
170.5520
171.5503
157.0941
254.0250
256.0223
258.0182
340.0980
351.0877
397.1660
445.4926
100
%
0
m/z
100 120 140 160 180 200 220 240 260 280 300 320 340 360 380 400 420 440 460 480

Compound：5

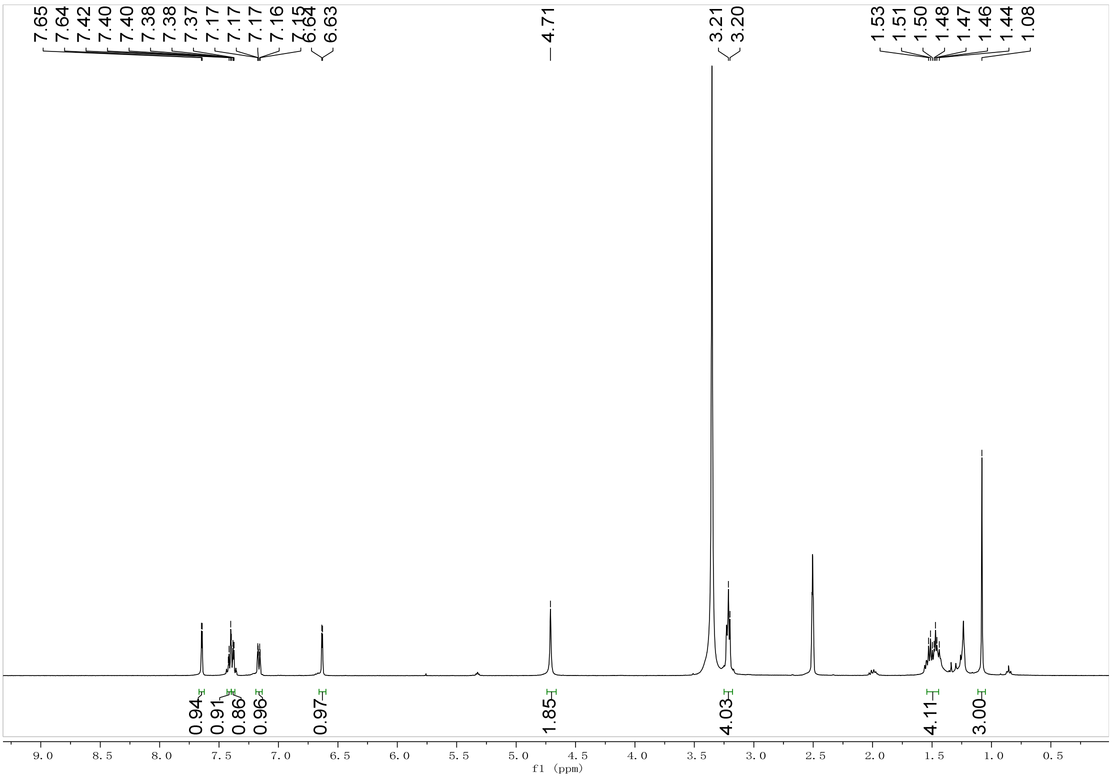

7.65 7.64 7.42 7.40 7.40 7.38 7.38 7.37 7.17 7.17 7.17 7.16 7.15 6.64 6.63
4.71
3.21 3.20
1.53 1.51 1.50 1.48 1.47 1.46 1.44 1.08
0.94 0.91 0.86 0.96 0.97 1.85 4.03 4.11 3.00
9.0 8.5 8.0 7.5 7.0 6.5 6.0 5.5 5.0 4.5 4.0 3.5 3.0 2.5 2.0 1.5 1.0 0.5
f1 (ppm)

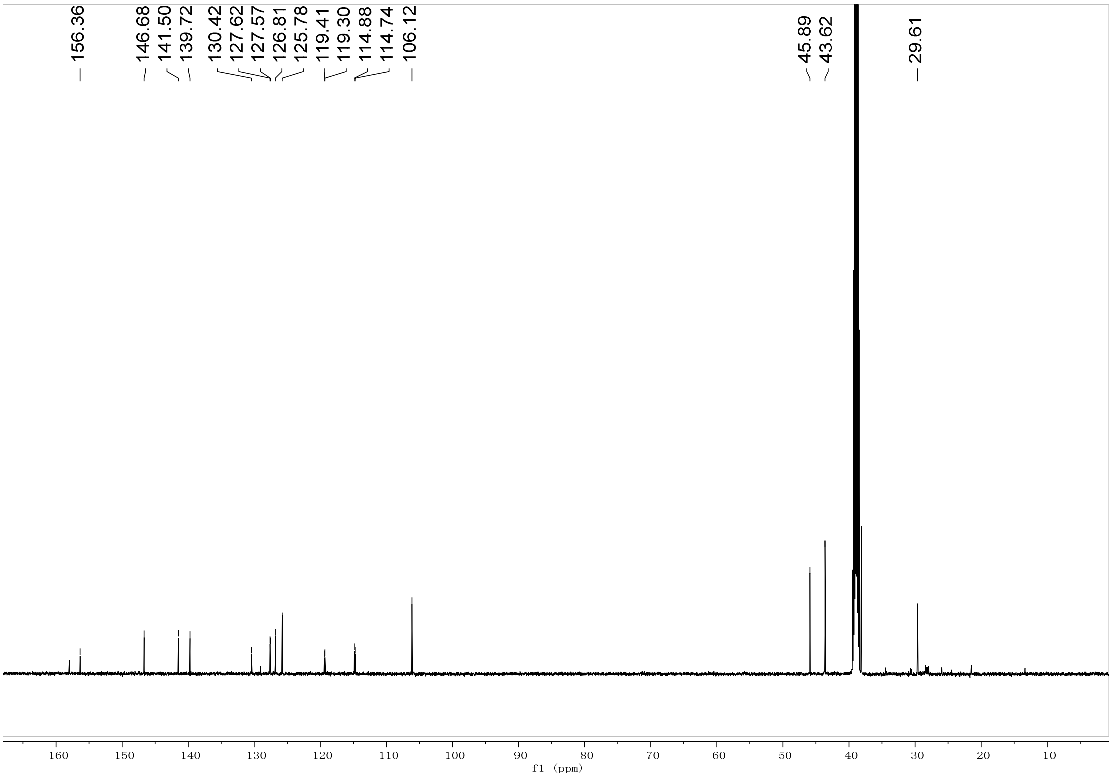

156.36
146.68 141.50 139.72
130.42 127.62 127.57 126.81 125.78 119.41 119.30 114.88 114.74 106.12
45.89 43.62
29.61
160 150 140 130 120 110 100 90 80 70 60 50 40 30 20 10
f1 (ppm)

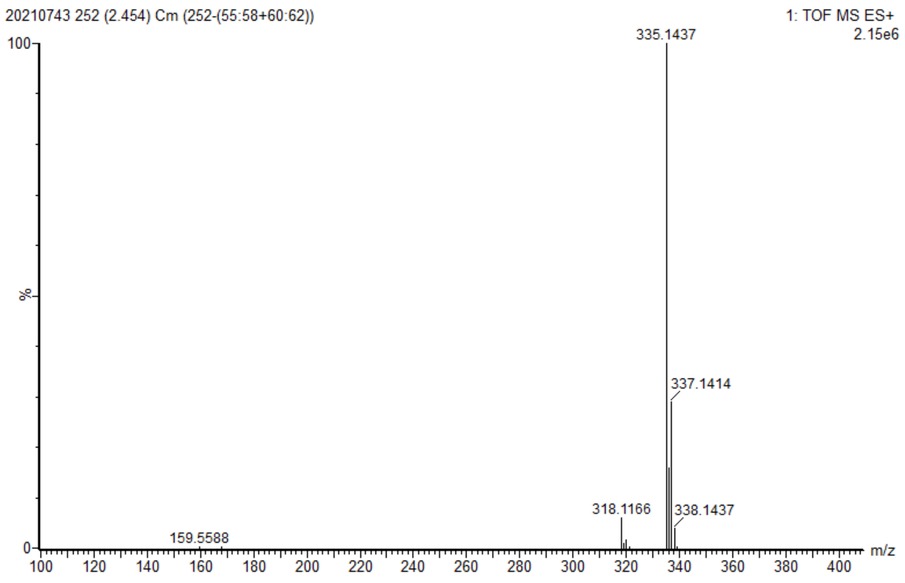
20210743 252 (2.454) Cm (252-(55:58+60:62))
1: TOF MS ES+
2.15e6
335.1437
337.1414
318.1166
338.1437
159.5588
100
%
0
m/z
100 120 140 160 180 200 220 240 260 280 300 320 340 360 380 400

Compound：6

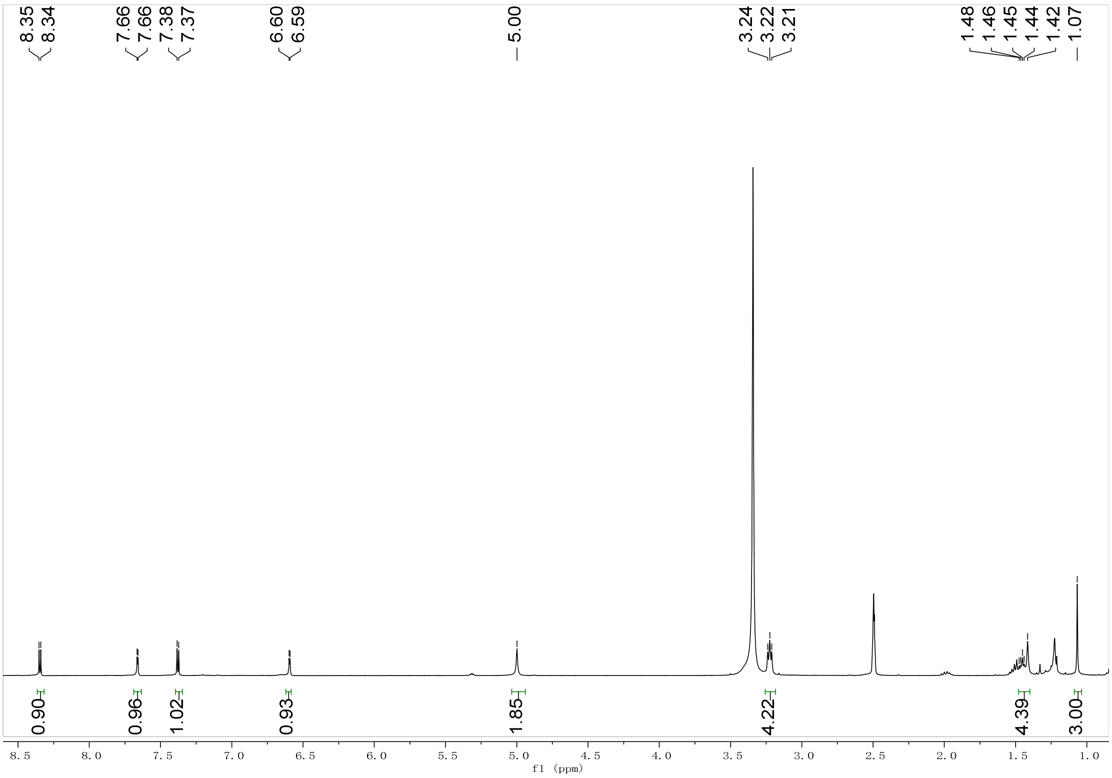
8.35 8.34 7.66 7.66 7.38 7.37 6.60 6.59 5.00 3.24 3.22 3.21 1.48 1.46 1.45 1.44 1.42 1.07
0.90 0.96 1.02 0.93 1.85 4.22 4.39 3.00
8.5 8.0 7.5 7.0 6.5 6.0 5.5 5.0 4.5 4.0 3.5 3.0 2.5 2.0 1.5 1.0
f1 (ppm)

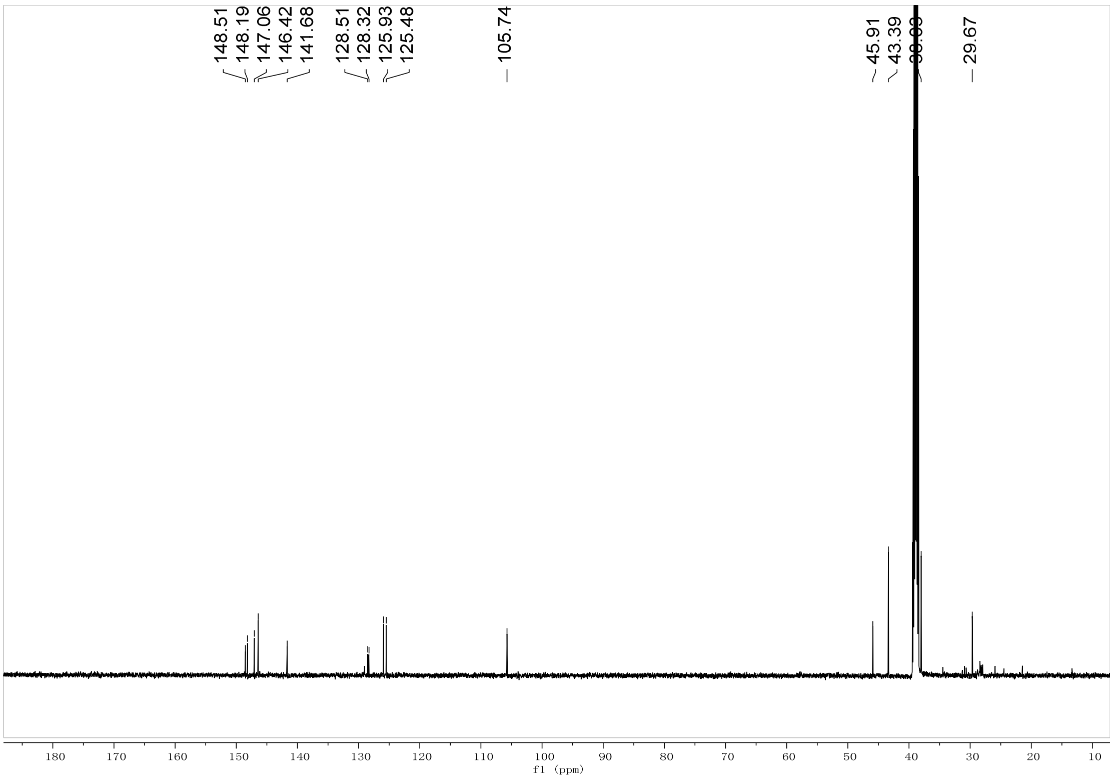
148.51
148.19
147.06
146.42
141.68
128.51
128.32
125.93
125.48
105.74
45.91
43.39
29.67
f1 (ppm)

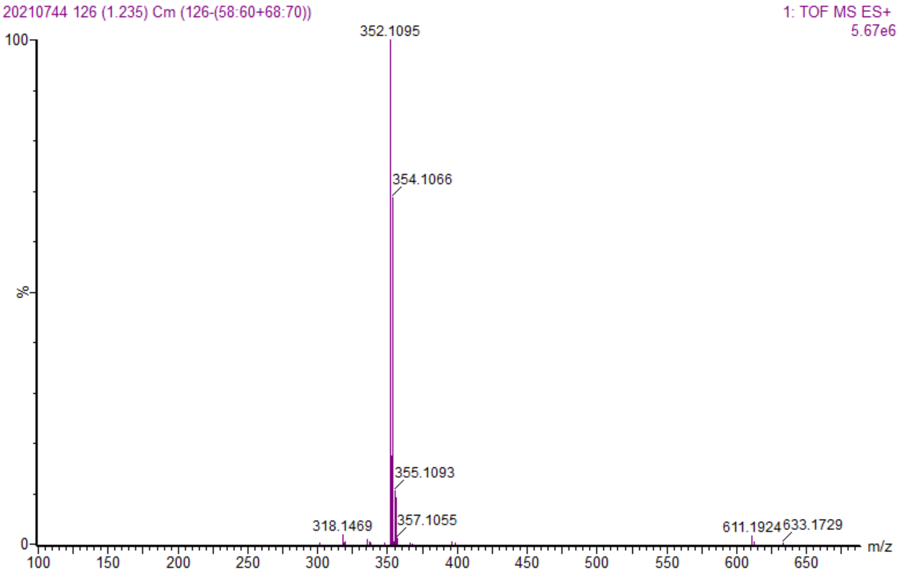
20210744 126 (1.235) Cm (126-(58:60+68:70))
1: TOF MS ES+
5.67e6
352.1095
354.1066
355.1093
357.1055
318.1469
611.1924
633.1729
%
m/z

Compound：7

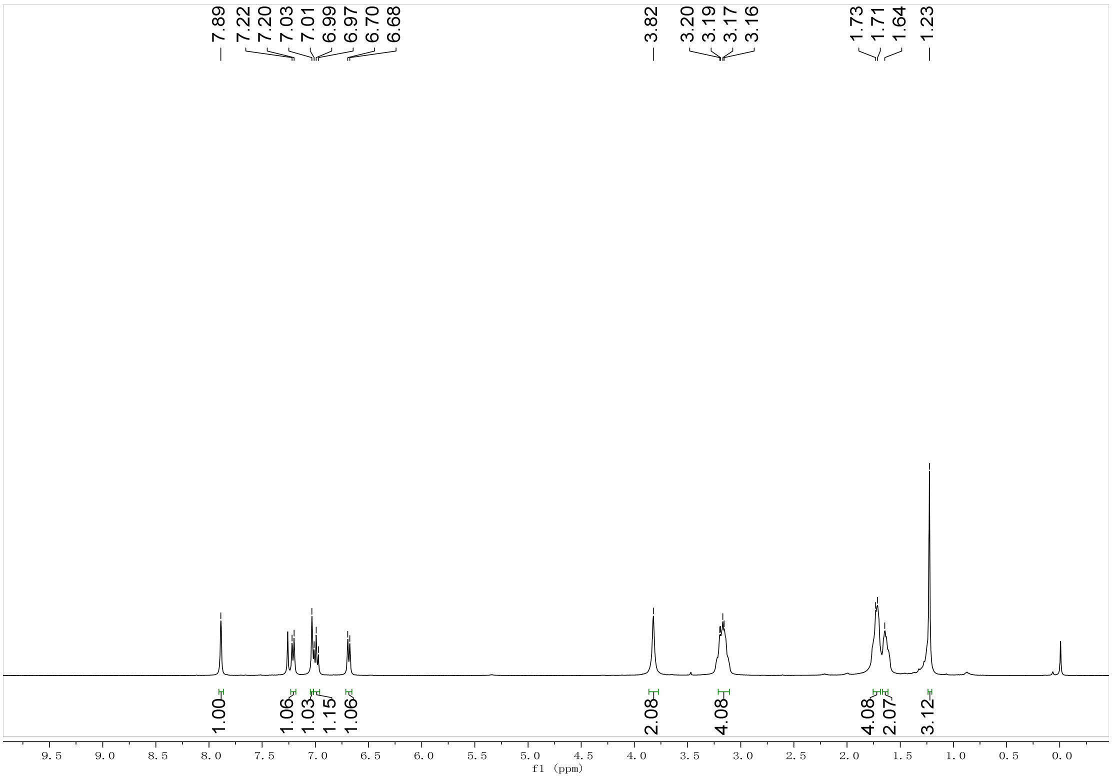
7.89
7.22
7.20
7.03
7.01
6.99
6.97
6.70
6.68
3.82
3.20
3.19
3.17
3.16
1.73
1.71
1.64
1.23
1.00
1.06
1.03
1.15
1.06
2.08
4.08
4.08
2.07
3.12
9.5 9.0 8.5 8.0 7.5 7.0 6.5 6.0 5.5 5.0 4.5 4.0 3.5 3.0 2.5 2.0 1.5 1.0 0.5 0.0
f1 (ppm)

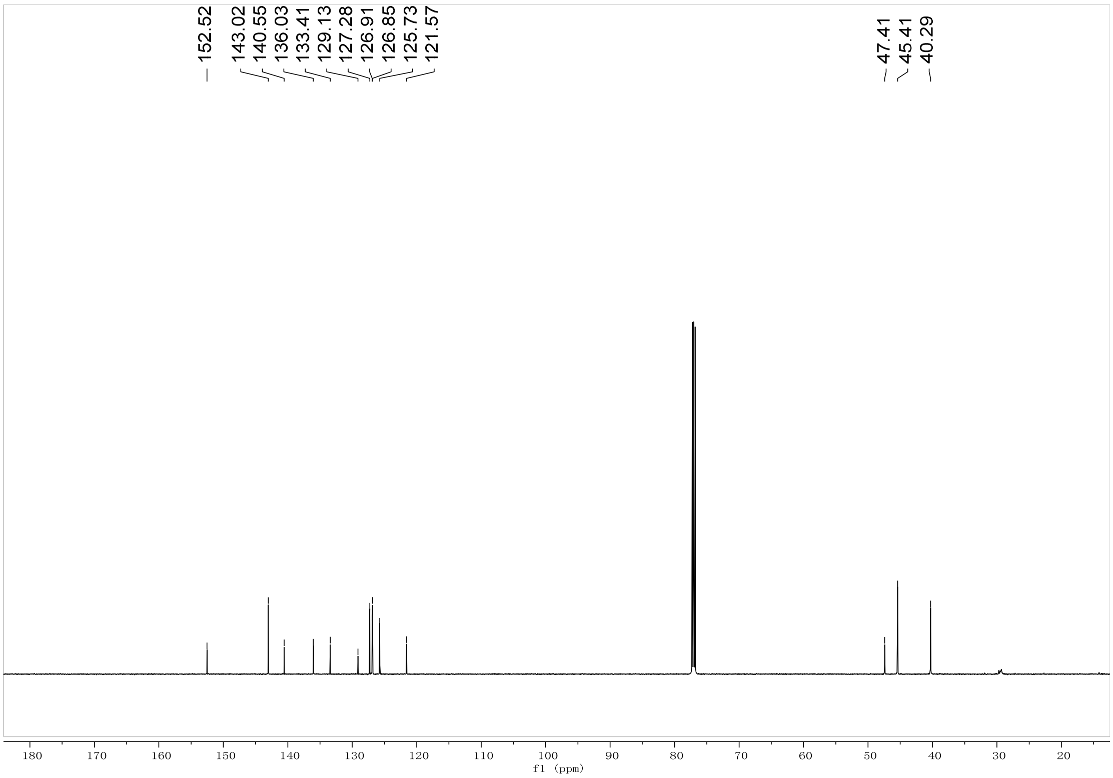
152.52
143.02
140.55
136.03
133.41
129.13
127.28
126.91
126.85
125.73
121.57
47.41
45.41
40.29
180 170 160 150 140 130 120 110 100 90 80 70 60 50 40 30 20
f1 (ppm)

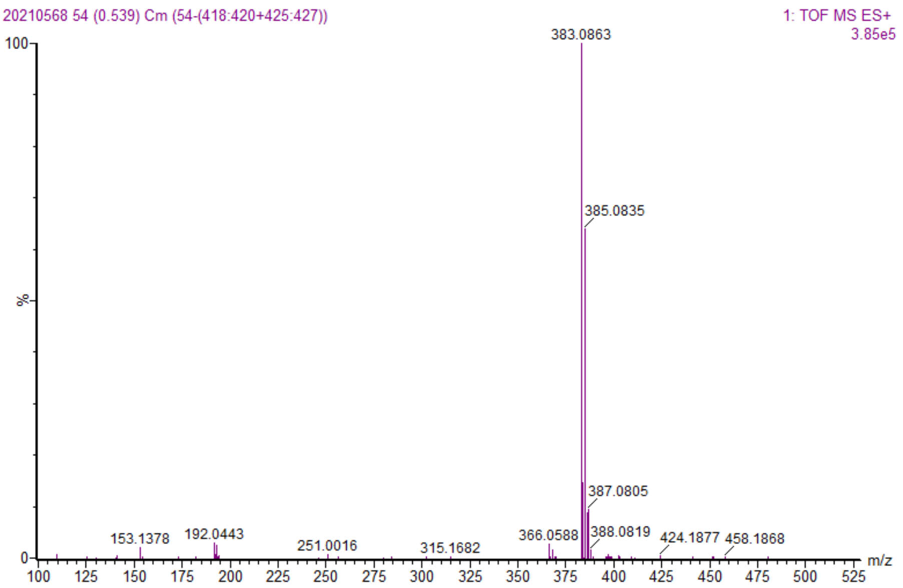
20210568 54 (0.539) Cm (54-(418:420+425:427))
1: TOF MS ES+
3.85e5
383.0863
385.0835
387.0805
388.0819
366.0588
424.1877
458.1868
153.1378
192.0443
251.0016
315.1682
%
m/z

Compound 8

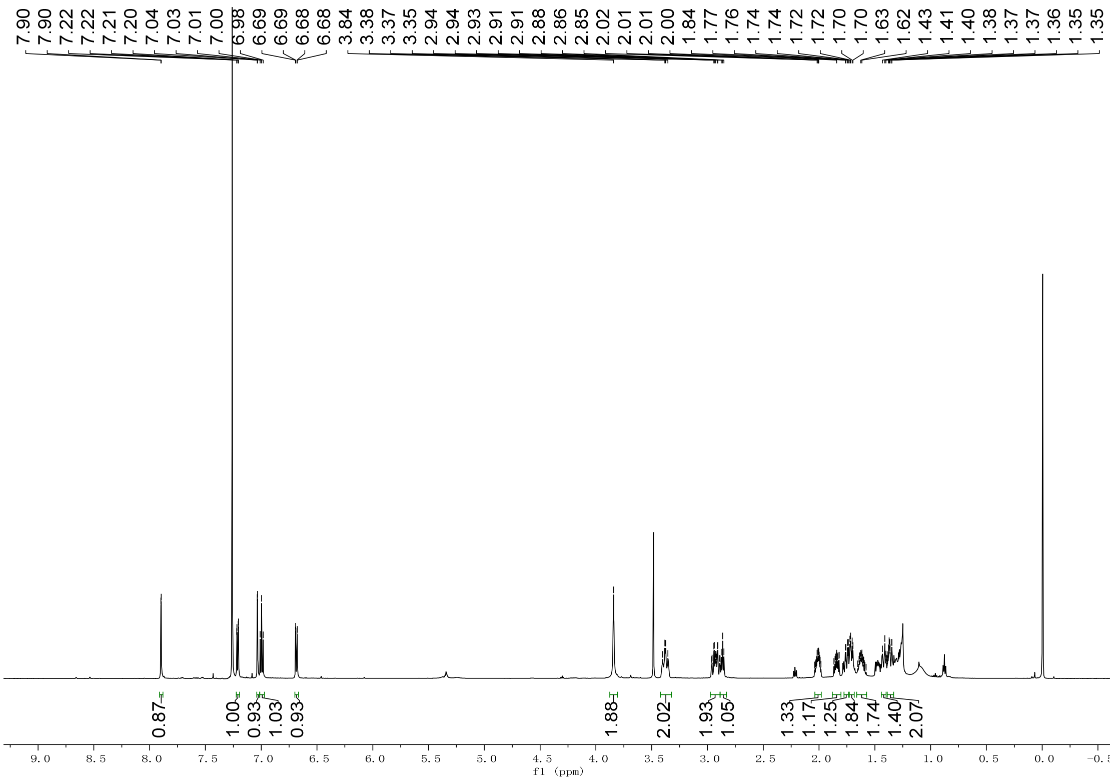
7.90 7.90 7.22 7.22 7.21 7.20 7.04 7.03 7.01 7.00 6.98 6.69 6.69 6.68 6.68 3.84 3.38 3.37 3.35 2.94 2.94 2.93 2.91 2.91 2.88 2.86 2.85 2.02 2.01 2.01 2.00 1.84 1.77 1.76 1.74 1.74 1.72 1.72 1.70 1.70 1.63 1.62 1.43 1.41 1.40 1.38 1.37 1.37 1.36 1.35 1.35
0.87 1.00 0.93 1.03 0.93 1.88 2.02 1.93 1.05 1.33 1.17 1.25 1.84 1.74 1.40 2.07
f1 (ppm)

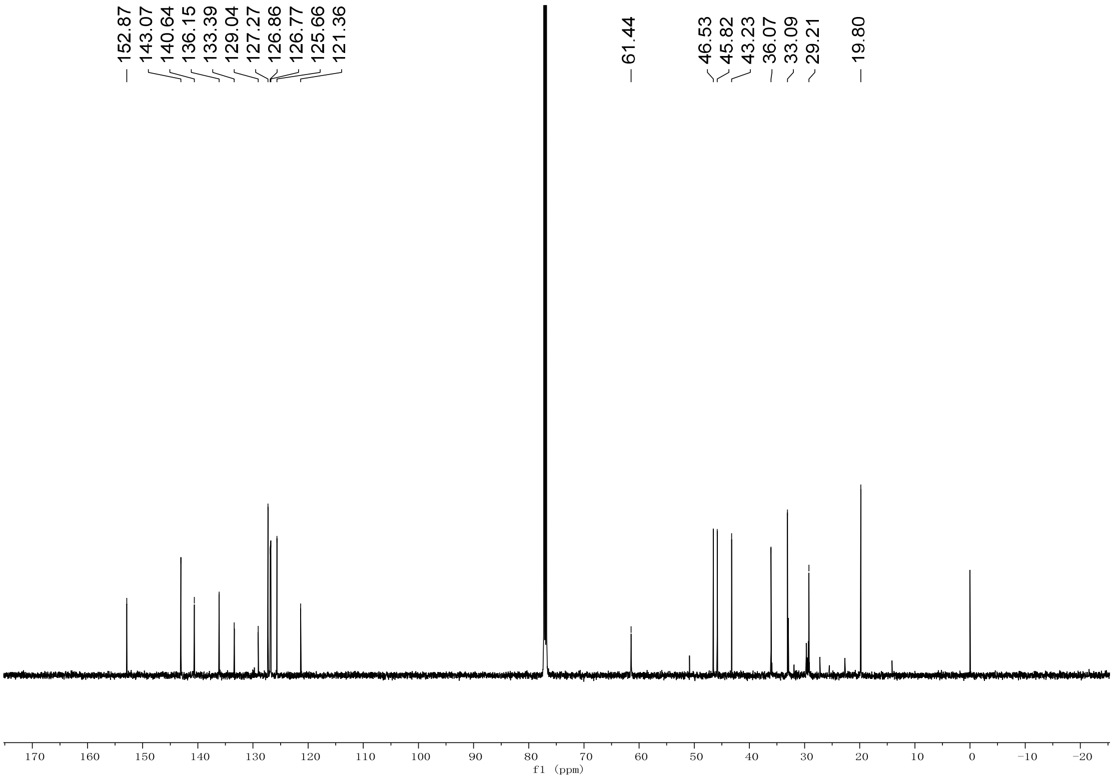
152.87
143.07
140.64
136.15
133.39
129.04
127.27
126.86
126.77
125.66
121.36
61.44
46.53
45.82
43.23
36.07
33.09
29.21
19.80
f1 (ppm)

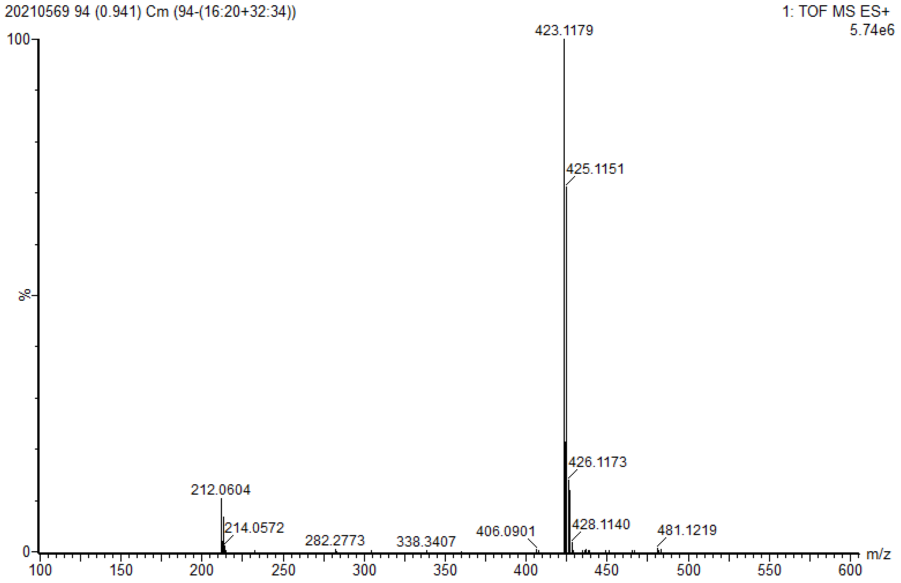
20210569 94 (0.941) Cm (94-(16:20+32:34))
1: TOF MS ES+
5.74e6
423.1179
425.1151
426.1173
212.0604
214.0572
282.2773
338.3407
406.0901
428.1140
481.1219
%
m/z
